# Exploring the butyrate metabolism-related shared genes in metabolic associated steatohepatitis and ulcerative colitis

**DOI:** 10.1038/s41598-024-66574-0

**Published:** 2024-07-10

**Authors:** Beiying Deng, Yinghui Liu, Ying Chen, Pengzhan He, Jingjing Ma, Zongbiao Tan, Jixiang Zhang, Weiguo Dong

**Affiliations:** 1 Department of Gastroenterology, Renmin Hospital of Wuhan University, No 99 Zhangzhidong Road, Wuhan, 430060 Hubei Province China; 2 Central Laboratory, Renmin Hospital of Wuhan University, Wuhan, China; 3 Department of Geriatric, Renmin Hospital of Wuhan University, Wuhan, China

**Keywords:** Metabolic associated fatty liver disease, Ulcerative colitis, Butyrate metabolism, Biomarkers, Machine learning, Genetics, Immunology

## Abstract

Metabolic-associated steatohepatitis (MASH) and ulcerative colitis (UC) exhibit a complex interconnection with immune dysfunction, dysbiosis of the gut microbiota, and activation of inflammatory pathways. This study aims to identify and validate critical butyrate metabolism-related shared genes between both UC and MASH. Clinical information and gene expression profiles were sourced from the Gene Expression Omnibus (GEO) database. Shared butyrate metabolism-related differentially expressed genes (sBM-DEGs) between UC and MASH were identified via various bioinformatics methods. Functional enrichment analysis was performed, and UC patients were categorized into subtypes using the consensus clustering algorithm based on sBM-DEGs. Key genes within sBM-DEGs were screened through Random Forest, Support Vector Machines-Recursive Feature Elimination, and Light Gradient Boosting. The diagnostic efficacy of these genes was evaluated using receiver operating characteristic (ROC) analysis on independent datasets. Additionally, the expression levels of characteristic genes were validated across multiple independent datasets and human specimens. Forty-nine shared DEGs between UC and MASH were identified, with enrichment analysis highlighting significant involvement in immune, inflammatory, and metabolic pathways. The intersection of butyrate metabolism-related genes with these DEGs produced 10 sBM-DEGs. These genes facilitated the identification of molecular subtypes of UC patients using an unsupervised clustering approach. ANXA5, CD44, and SLC16A1 were pinpointed as hub genes through machine learning algorithms and feature importance rankings. ROC analysis confirmed their diagnostic efficacy in UC and MASH across various datasets. Additionally, the expression levels of these three hub genes showed significant correlations with immune cells. These findings were validated across independent datasets and human specimens, corroborating the bioinformatics analysis results. Integrated bioinformatics identified three significant biomarkers, ANXA5, CD44, and SLC16A1, as DEGs linked to butyrate metabolism. These findings offer new insights into the role of butyrate metabolism in the pathogenesis of UC and MASH, suggesting its potential as a valuable diagnostic biomarker.

## Introduction

Inflammatory bowel disease (IBD) and metabolic-associated fatty liver disease (MAFLD) present a significant global burden, impacting millions of individuals worldwide and leading to substantial morbidity, mortality, and healthcare costs^1,2^. In recent decades, MAFLD has become the most prevalent chronic liver disorder, with an estimated global adult prevalence of approximately 25%^3^. Simultaneously, IBD has remained a common chronic gastrointestinal condition, with both its incidence and prevalence continuing to increase^4,5^.

Despite the elusive precise common pathogenesis and causal relationship between MAFLD and IBD, substantial evidence supports potential physiological and pathological interconnections between these conditions^6–8^. Variations in results from diverse population data and multicenter studies notwithstanding, research consistently indicates a link between IBD and MASH, a more severe form of MAFLD^9^. Specifically, the incidence rates of MASH development in IBD patients and the incidence rates of IBD development in MASH patients are significantly higher than those in the general population^10^. This trend persists even when considering statistically significant variations in incidence rates or risk ratios for MASH development between Crohn's disease (CD) and ulcerative colitis (UC) patients^11,12^. Additionally, evidence suggests that MASH mice induced by a high-fat diet are more susceptible to colitis development, resulting in notably higher mortality rates compared to the control group^13^. Comprehensive reviews have summarized shared risk factors between MAFLD and IBD, such as obesity, insulin resistance, and metabolic syndrome. Other contributing factors may include systemic inflammation, the use of IBD therapeutic drugs with potential hepatotoxicity, gut microbiota dysbiosis, bile acid metabolism, and intestinal barrier dysfunction^9,14,15^. However, despite this existing knowledge, the underlying mechanisms linking IBD and MASH comorbidity remain unclear, posing a significant challenge in managing patients with this combined condition.

The role of butyrate in linking IBD and MAFLD has garnered significant attention due to its potential impact on dysregulated gut microbiota and its metabolites, which lead to inflammation and immune infiltration in both conditions^16^. Butyrate, a short-chain fatty acid, has shown beneficial effects in managing UC by promoting colonic motility, enhancing intestinal blood flow, improving intestinal mucosal barrier function, and modulating immune responses^17–19^. Additionally, butyrate demonstrates anti-inflammatory effects in MASH by preserving gut barrier function, regulating gut microbiota, modulating lipid metabolism, and alleviating insulin resistance^20,21^. These combined effects suggest that butyrate may play a crucial role in mitigating the common pathophysiology of UC and MASH.

Investigating the pathways of butyrate metabolism in both UC and MASH is therefore essential for understanding their common pathogenesis and clinical implications. This study identified ANXA5, CD44, and SLC16A1 as critical genes related to butyrate metabolism and explored their correlation with immune infiltration. The results were confirmed in the validation cohort and further validated through human samples. Notably, this is the first study to elucidate the common genetic characteristics and molecular mechanisms between UC and MASH, offering a new perspective on the intrinsic link between these two conditions.

## Methods

### GEO dataset selection

Colonic mucosa biopsy samples from UC patients and liver tissue samples from MASH patients were obtained from the GEO database (http://www.ncbi.nlm.nih.gov/geo). After removing outlier samples and genes with low expression and abundance, the "ComBat" algorithm from the "sva" R package was applied to minimize batch effects^22^. By integrating datasets GSE61260 and GSE63067, a consolidated cohort named "MASH MergeCo" was established. For a more comprehensive exploration of the mechanistic interplay between MASH and UC, the independent validation sets GSE213621 and GSE87466 were identified. Additionally, a comprehensive collection of 882 butyrate metabolism-related genes with relevance scores greater than 5 was compiled from GeneCards (https://www.genecards.org).

All liver biopsy specimens were staged according to the published NASH Clinical Research Network scoring system^23^. For the analyses, fibrosis stages 1a, 1b, and 1c were combined and treated as stage 1 fibrosis.

### Weighted gene co-expression network analysis (WGCNA)

The "WGCNA" R package facilitated WGCNA and the identification of gene modules associated with UC^24^. This process involved assessing scale independence and average connectivity across various power values using the "pickSoftThreshold" function, ultimately selecting the optimal soft threshold *β*. Hierarchical clustering grouped genes with similar expression patterns into cohesive modules. Dynamic tree cutting segmented and recognized co-expression modules, followed by merging modules with similar expression profiles. Pearson correlation analysis was then performed to determine the relationship between modules and clinical attributes.

### Identification and enrichment analyses of differentially expressed genes (DEGs)

DEGs were evaluated using Wilcoxon's rank-sum test, and potential shared genes were identified through Venn diagrams. An intersection analysis of butyrate metabolism-related genes with UC-related modules, UC-DEGs, and MASH-DEGs was performed, defining the resulting genes as shared butyrate metabolism-related DEGs (sBM-DEGs). Gene Ontology (GO) and Kyoto Encyclopedia of Genes and Genomes (KEGG) enrichment analyses were conducted to gain a comprehensive understanding of the biological mechanisms underpinning the identified DEGs^25^. These analyses utilized the R packages "clusterProfiler" and "org.Hs.eg.db" with a set threshold of q-value 0.05 to ensure robust results^26^.

### Assessment of immune infiltration patterns

Enrichment levels of immune cell infiltration were computed using the ssGSEA method^27^. The Student's t-test validated distinctions between the two groups, and results were graphically depicted using R's "ggboxplot" package. The "corrplot" package investigated the relationship between hub genes and immune cells, while the "pheatmap" package visualized the findings.

### Identification of transcription factors (TFs)

Information from the STRING database (https://string-db.org) was used to create a human protein–protein interaction (PPI) network. The MCC algorithm was then applied using the cytoHubba plug-in in Cytoscape (version 3.9.1) to identify the top ten hub genes within the PPI network. The Transcriptional Regulatory Relationships Unraveled by Sentence-based Text Mining (TRRUST) v2 database (https://www.grnpedia.org/trrust/) provided regulatory insights into TFs and their target genes. A network demonstrating TF-target interactions was created using the discovered TF-target regulatory linkages.

### Unsupervised clustering and functional enrichment analysis of patients with MASH

The "ConsensusClusterPlus" package was employed for unsupervised clustering analysis^28^. The k-means algorithm was used with a maximum subtype number of k (k = 6), completing 1,000 iterations. The ideal subtype number was determined based on the analysis of the cumulative distribution function (CDF) curve, consensus matrix, and consistent cluster score.

The "GSVA" package was utilized to perform a GSVA enrichment analysis to compare the enriched pathways and functionalities of various butyrate metabolism-related subtypes^29^. Two gene sets from the MSigDB database, "c2.cp.kegg.v7.4.symbols" and "c5.go.bp.v7.5.1.symbols," were used as input files. By comparing the GSVA scores between various subtypes, differential enrichment functions and pathways were identified using the "limma" R package. Functions and pathways with a |t| value of the GSVA score greater than two were considered notably enhanced.

### Identification of signature genes by machine learning

Potential biomarkers were identified by further screening the sBM-DEGs using three machine learning algorithms: Light Gradient Boosting Model (LightGBM), Support Vector Machine-Recursive Feature Elimination (SVM-RFE), and Random Forest (RF). The "DALEX" package facilitated model interpretation, enabling the calculation of both local and global feature importance to enhance model understanding. The predictive accuracy of the feature genes identified by these machine-learning techniques was evaluated using the validation set, with results visually represented by ROC curves.

### Immunohistochemistry (IHC)

Colon biopsies were obtained from patients with UC and healthy controls (HC), while liver biopsies were derived from patients with MASH and HC. Samples were sourced from the Department of Pathology, Renmin Hospital of Wuhan University. Paraffin-embedded slices underwent deparaffinization and rehydration using slide warmers and alcohol. Antigen retrieval was performed with citrate buffer for 60 min. To inhibit endogenous peroxidase activity, all sections were treated with 0.3% hydrogen peroxide for 10 min. A blocking step with 5% bovine serum albumin (BSA) was conducted before coating sections with primary antibodies, followed by overnight incubation at 4 °C. After washing, sections were incubated with a secondary antibody for 20 min at room temperature. Diaminobenzidine (DAB) staining was carried out using a DAB kit (Servicebio), and sections were counterstained with hematoxylin. Stained sections were examined using light microscopy. Primary antibodies targeting ANXA5 (Cat No. 11060-1-AP), CD44 (Cat No. 15675-1-AP), and SLC16A1 (Cat No. 20139-1-AP) were used.

The study adhered to the Declaration of Helsinki and received approval from the Ethics Committee of Renmin Hospital of Wuhan University (WDRY2023-K151 and WDRY 2022-K130). Informed consent was obtained from all patients, and the research was conducted in accordance with relevant guidelines and regulations.

### Statistical analysis

All statistical analyses for this study were conducted using R (version 4.2.3) and GraphPad Prism (version 8.3.0). Statistical significance was defined as a *p*-value or adjusted *p*-value of less than 0.05, based on two-sided tests.

## Result

### Identification of DEGs between UC patients and HC

**Figure 1:**
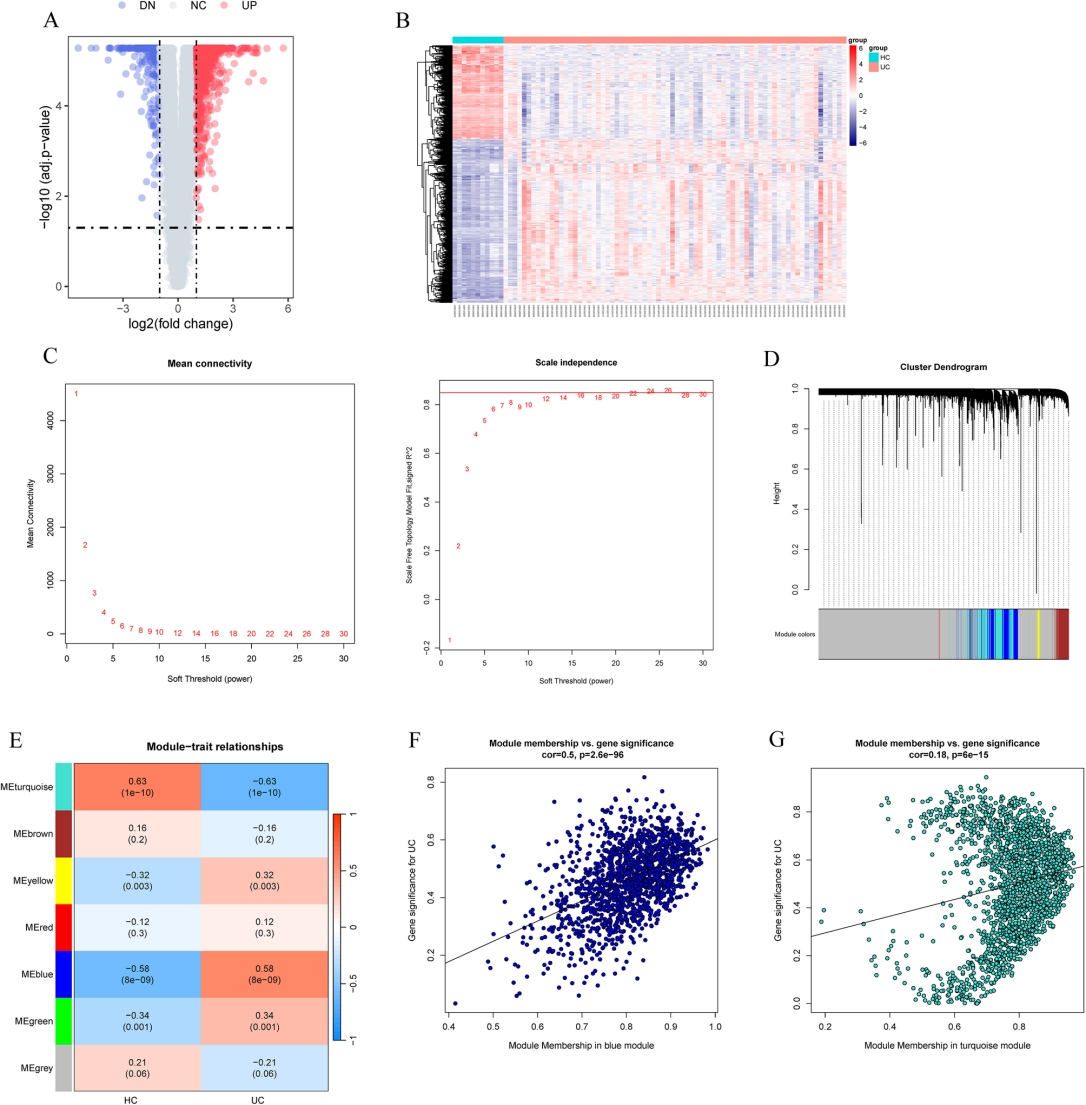
(**A**) Volcano plot illustrating DEGs in colon tissue between UC patients and HC. (**B**) Heatmap depicting gene expression profiles in UC patients and HC. (**C**) Analysis of scale-free index and mean connectivity for soft-threshold power determination. (**D**) Module clustering dendrogram based on dissimilarity measure (1-TOM). (**E**) Heatmap revealing a correlation between module eigengenes and UC. (**F**, **G**) Scatter plot of the GS for UC vs. the MM in the blue (**F**) and turquoise (**G**) modules. DEG, differentially expressed genes. UC, ulcerative colitis. HC, healthy controls. TOM, topological overlap matrix. GS, gene significance. MM, module membership.

The UC microarray dataset GSE75214 was obtained from the GEO database. Using criteria of an adjusted *p*-value of 0.05 and an absolute mean difference greater than 1, a total of 1124 DEGs were identified, with 715 upregulated and 409 downregulated (Fig. 1A,B). All samples from GSE75214 underwent clustering and were included in the WGCNA. An optimal soft threshold power *β* of 24 was determined, yielding a scale-free R^2^ of 0.85 (Fig. 1C). Dynamic tree cutting revealed 7 distinct modules after merging those with similar expression profiles (Fig. 1D,E). Among these, the turquoise and blue modules exhibited the most significant correlation with UC (Fig. 1E–G) (Table S1).

### Identification of shared DEGs between MASH and HC

**Figure 2:**
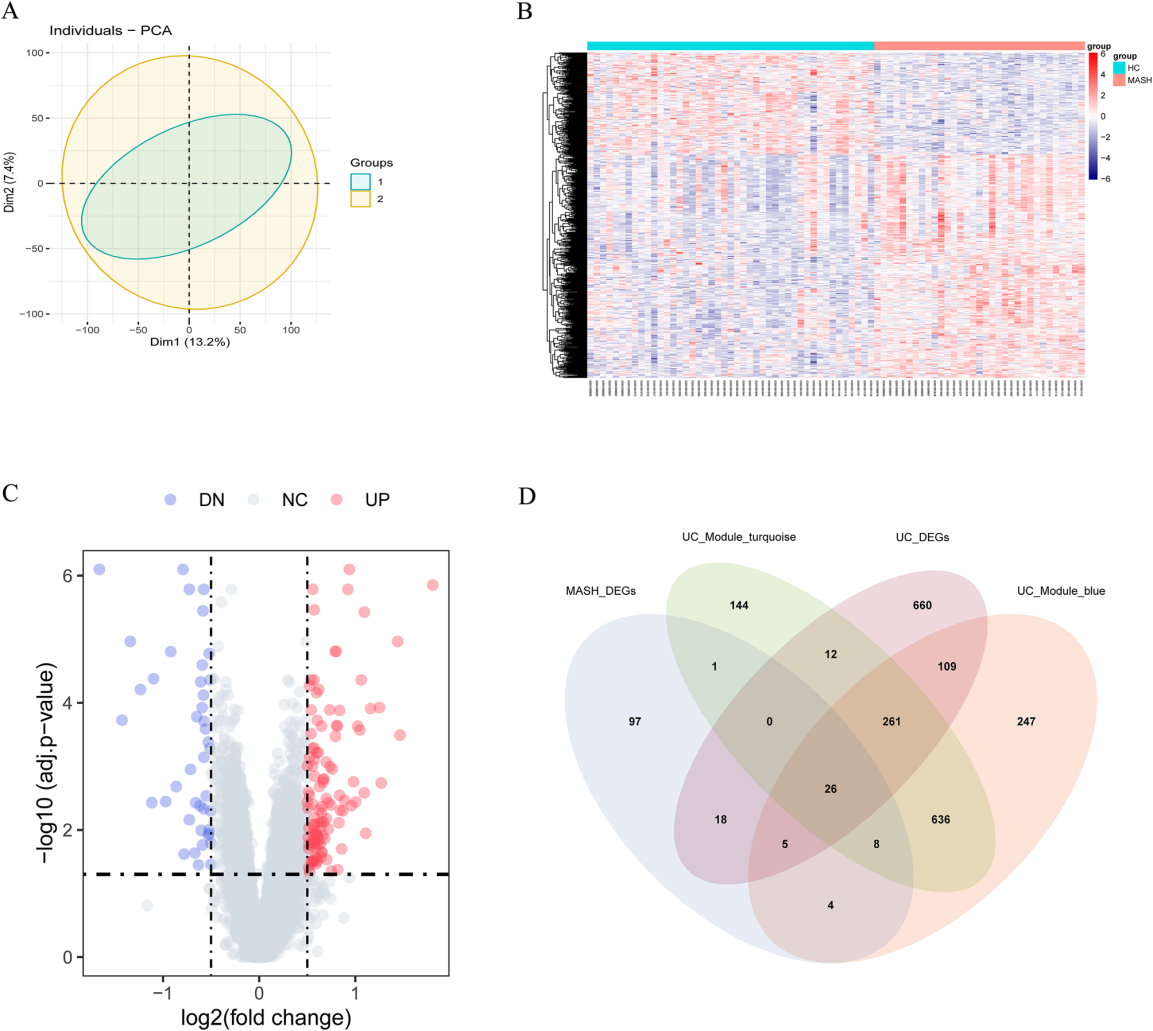
(**A**) PCA of two datasets after batch effect correction. (**B**) Heatmap depicting gene expression profiles in MASH patients and HC. (**C**) Volcano plot showcasing DEGs in liver tissue between MASH patients and HC. (**D**) Venn diagram indicating overlapping genes among MASH-DEGs, UC-DEGs, and UC-related modules.

The principal component analysis plot of the MASH MergeCo successfully distinguished between the MASH patients and HC, confirming effective batch effect correction (Fig. 2A). Comparing HC to MASH patients revealed 159 DEGs, with Benjamini–Hochberg adjusted *p*-values of 0.05 and an absolute mean difference greater than 0.5, including 117 upregulated and 42 downregulated DEGs (Fig. 2B,C). Additionally, Venn diagram analysis identified 49 shared DEGs among MASH-DEGs, UC-DEGs, and the UC-related module (Fig. 2D) (Table S2).

### Functional enrichment analysis and identified butyrate metabolism-related shared DEGs

**Figure 3:**
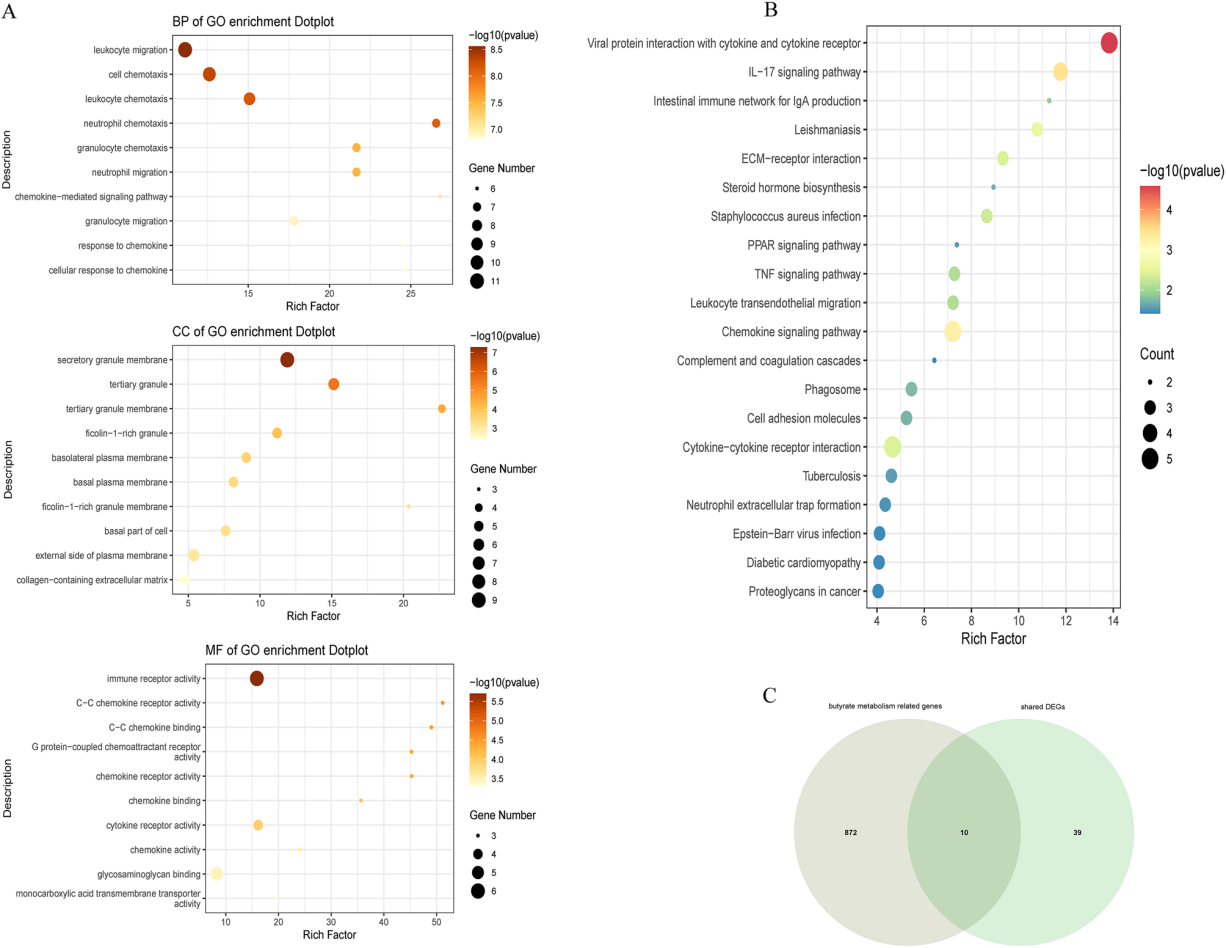
(**A**) GO enrichment analysis of shared DEGs. (**B**) KEGG pathway enrichment analysis of shared DEGs. (**C**) Venn diagram illustrating overlapping genes between butyrate metabolism-related genes and shared DEGs. GO, Gene Ontology. KEGG, Kyoto Encyclopedia of Genes and Genomes.

Functional enrichment analysis was conducted to elucidate the putative biological functions of the shared DEGs. GO analysis revealed high enrichment in processes related to immune receptor activity, C–C chemokine receptor activity, cell chemotaxis, and leukocyte chemotaxis (Fig. 3A). Additionally, KEGG analysis highlighted shared DEGs connected to inflammatory pathways, including the chemokine signaling pathway, IL-17 signaling pathway, intestinal immune network for IgA production, ECM-receptor interaction, PPAR signaling pathway, and TNF signaling pathway (Fig. 3B).

Gut microbiota alterations are thought to play a role in the disease process of IBD combined with MAFLD^30^. IBD and MAFLD displayed similar changes in gut microbiota composition^31^. Changes in the composition of the intestinal flora in IBD patients lead to changes in the microbial metabolites, such as butyric acid, which can further aggravate damage to the intestinal barrier and thus may affect the liver through leakage through the damaged intestinal barrier, leading to MAFLD^31^. Given the pivotal role of the butyrate metabolic pathway in both MASH and UC pathogenesis, its mechanistic involvement in the co-pathogenesis of UC and MASH was investigated. By intersecting butyrate metabolism-related genes with the shared DEGs, a set of 10 sBM-DEGs was identified (Fig. 3C).

### Identification of TFs

**Figure 4:**
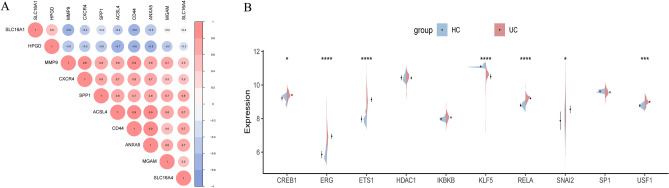
(**A**) Correlation plot of sBM-DEGs. (**B**) Split violin plot displaying transcription factor expression levels.

The preceding analysis revealed a mutual correlation among all 10 sBM-DEGs (Fig. 4A). Given that co-expressed genes often share common regulatory TFs, a TF enrichment analysis was conducted using the TRRUST database to identify key regulatory genes. This analysis identified SNAI2, ERG, IKBKB, HDAC1, ETS1, KLF5, USF1, CREB1, SP1, and RELA as the top 10 significantly enriched TFs among the 10 sBM-DEGs. Notably, elevated colonic expression of SNAI2, ERG, ETS1, USF1, CREB1, and RELA was observed. In contrast, KLF5 expression was lower in UC patients compared to HC (Fig. 4B).

### Development of UC molecular subtypes using unsupervised clustering

**Figure 5:**
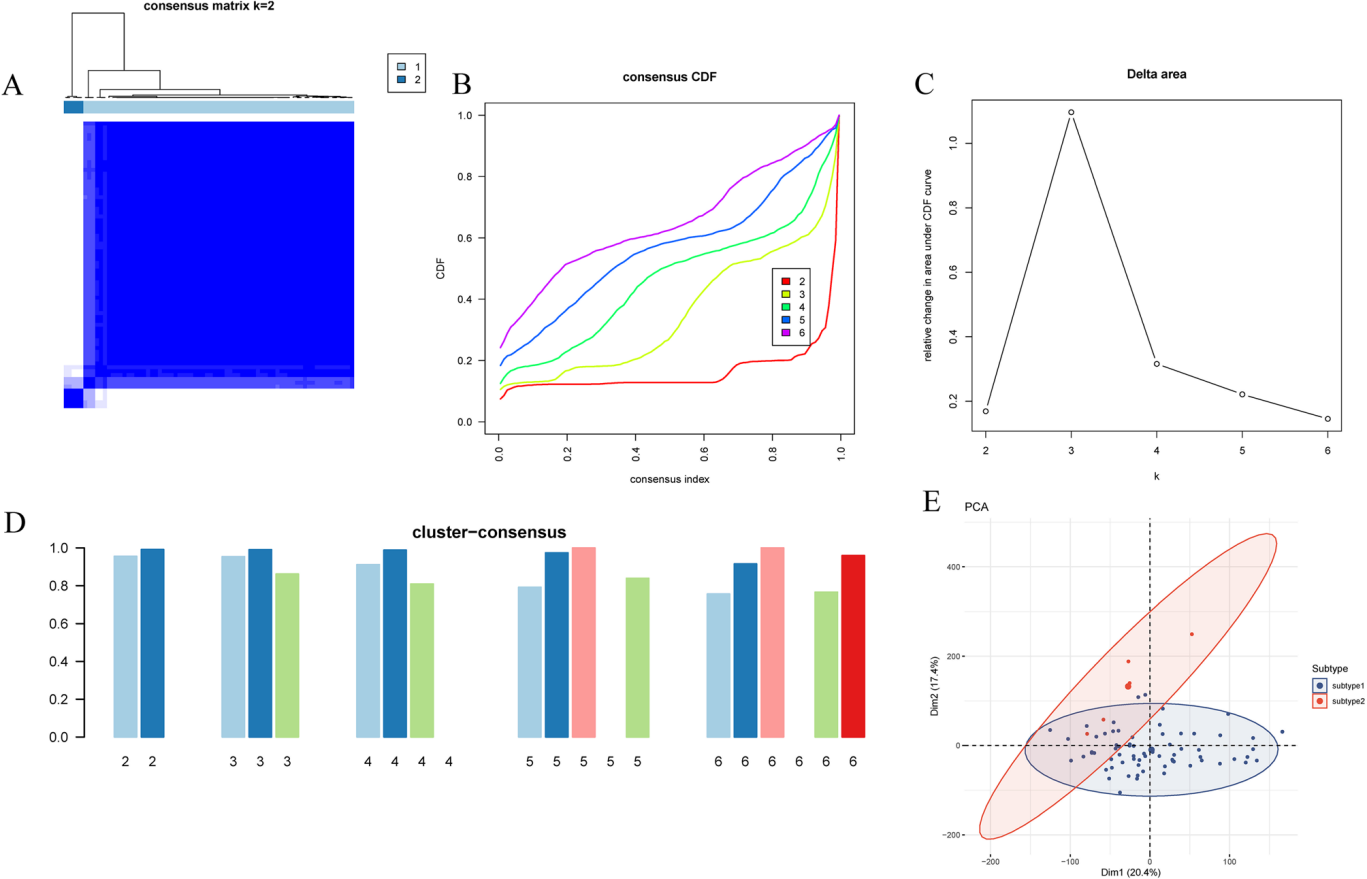
(**A**) Consensus clustering matrix for k = 2. (**B**) Consensus CDF curves for k = 2 to 6. (**C**) Relative alterations in CDF delta area curves. (**D**) Consensus score of each subtype for k = 2 to 6. (**E**) PCA diagram separating subtype 1 (blue) and subtype 2 samples (red). CDF, cumulative distribution function. PCA, principal component analysis.

To illustrate the butyrate metabolism-related patterns of UC, an unsupervised cluster analysis was conducted on 74 UC patients using the "ConsensusClusterPlus" R package, based on the expression patterns of 10 sBM-DEGs. Setting the cluster number to 2 yielded subgroup consistency scores greater than 0.8, supported by stable results from the CDF plot, delta area plot, tracking plot, and consensus matrix (Fig. 5A–D). Consequently, the 74 UC patients were categorized into two distinct clusters: molecular subtype 1 (n = 69) and molecular subtype 2 (n = 5) (Fig. 5E).

### Identification of immune microenvironment and biological function characteristics in different subtypes

**Figure 6:**
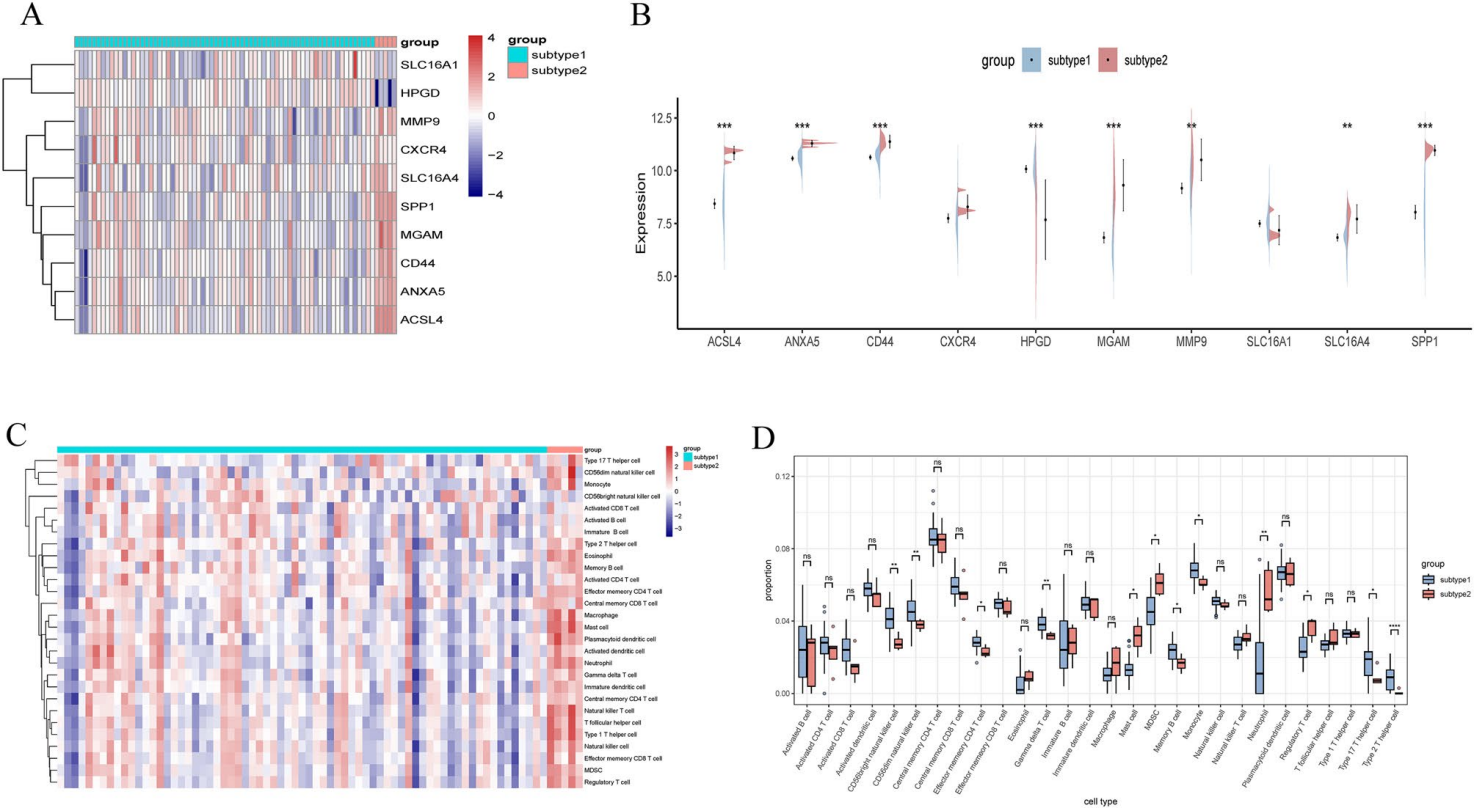
(**A**, **B**) Heatmap (**A**) and split violin plot (**B**) revealing the expression of 10 sBM-DEGs between subtypes. (**C**) Heatmap presenting diverse infiltrated immune cell abundances between subtypes. (**D**) Boxplot demonstrating immune cell differences between subtypes.

Differences in the expression of core regulators were evaluated to better understand the molecular characteristics between subtypes. Results indicated significant up-regulation of ACSL4, ANXA5, CD44, MGAM, MMP9, SLC16A4, and SPP1 in subtype 2, alongside notable down-regulation of HPGD (Fig. 6A,B). Additionally, substantial differences in immune cell infiltration quantities were observed between subtypes 1 and 2 (Fig. 6C). Specifically, subtype 1 exhibited increased amounts of CD56bright, CD56dim, effector memory CD4 T cells, γ&δ T cells, memory B cells, monocytes, type 17 T helper cells, and type 2 T helper cells. In contrast, subtype 2 showed a higher abundance of activated mast cells, MDSCs, and neutrophils (Fig. 6D).

**Figure 7:**
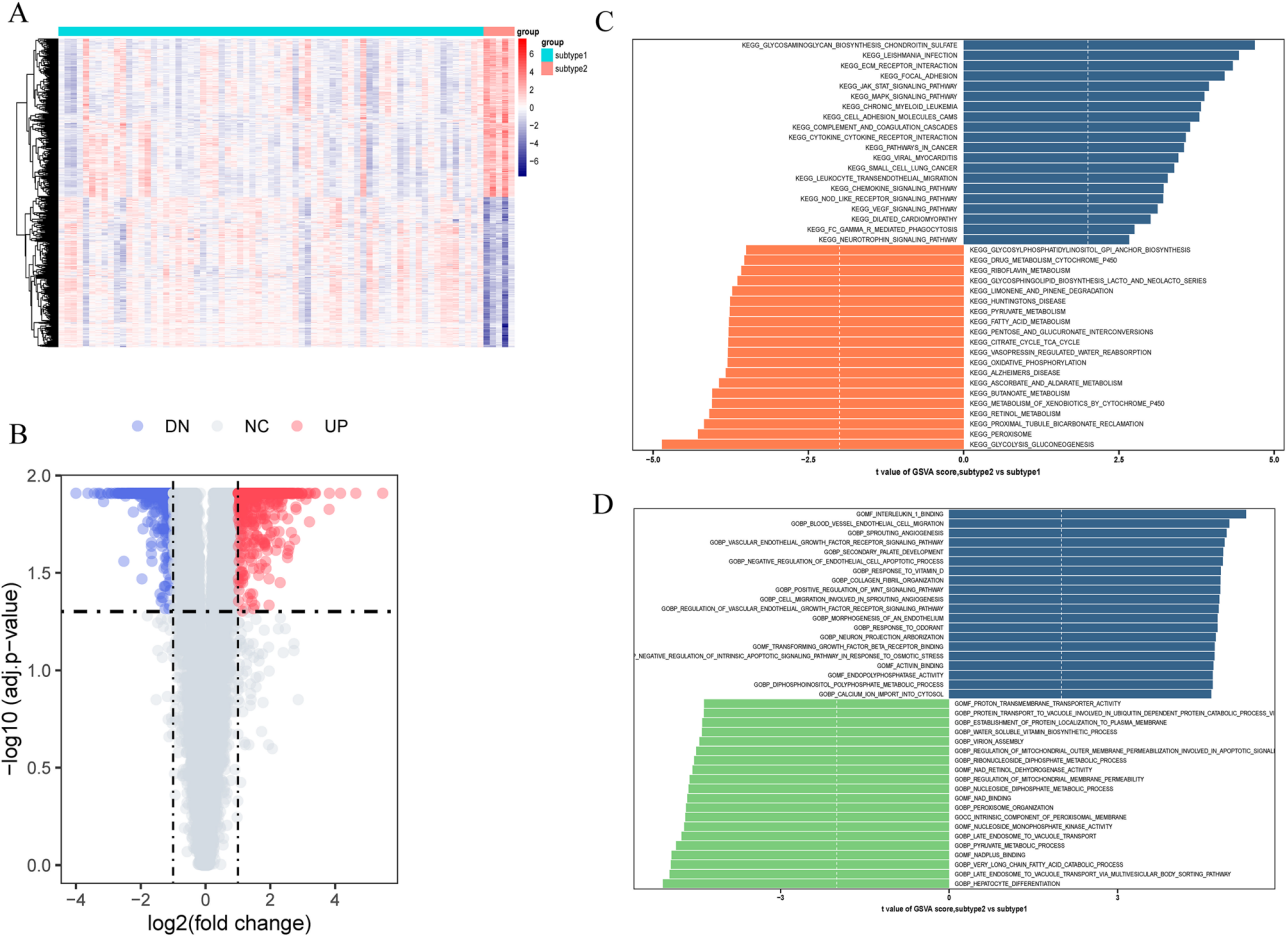
(**A**, **B**) Heatmap (**A**) and volcano plot (**B**) revealing the gene expression patterns between the two subtypes. (**C**, **D**) Differences in enriched biological functions (**C**) and hallmark pathways (**D**) between distinct immune microenvironment subtypes ranked by t values of GSVA scores. GSVA, gene-set variation analysis.

Significant differences in gene expression patterns between the two subtypes were observed (Fig. 7A,B). To assess variations in functional pathways linked to the distinct UC subtypes, GSVA was performed. The GSVA results revealed notable upregulation of processes such as very long chain fatty acid catabolic processes, pyruvate metabolic processes, late endosome to vacuole transport, peroxisome organization, and regulation of mitochondrial outer membrane permeabilization and inner membrane permeability in subtype 1 (Fig. 7C). Additionally, KEGG analysis of the GSVA results demonstrated upregulation of pathways including glycolysis gluconeogenesis, peroxisome, butanoate metabolism, ascorbate and aldarate metabolism, and oxidative phosphorylation in subtype 1 (Fig. 7D).

### Identification of hub diagnostic gene by machine-learning algorithms

**Figure 8:**
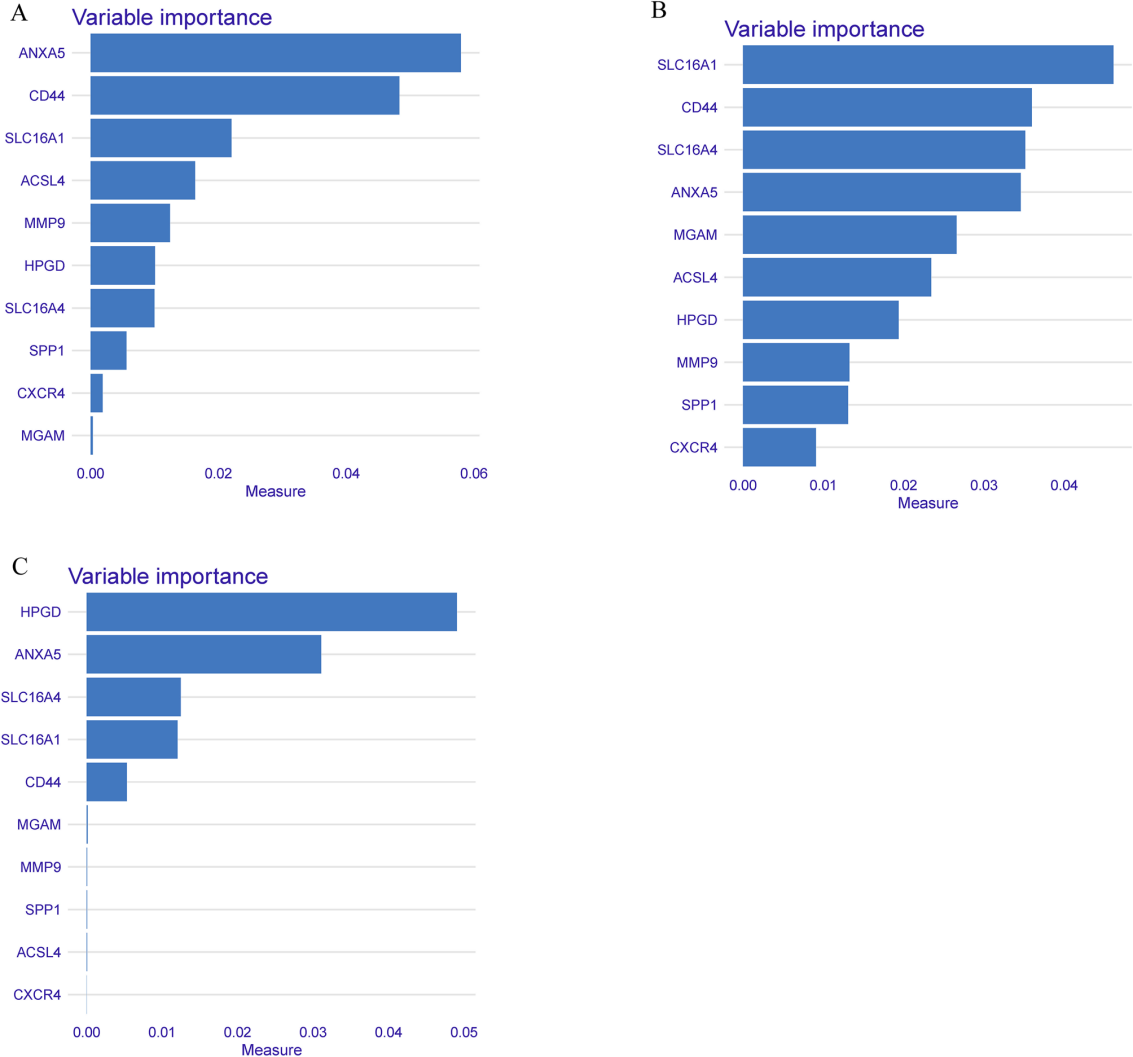
(**A**–**C**) Variable importance plot in machine learning: Random Forest (**A**), SVM-RFE (**B**), and LightGBM (**C**).

The selection of diagnostic genes was refined using three machine-learning techniques: LightGBM, SVM-RFE, and RF. By analyzing variable importance, characteristic genes were finalized based on the consensus of the LightGBM, SVM-RFE, and RF algorithms. ANXA5, CD44, and SLC16A1 were identified as the top three hub genes (Fig. 8).

### Validation of hub diagnostic genes

**Figure 9:**
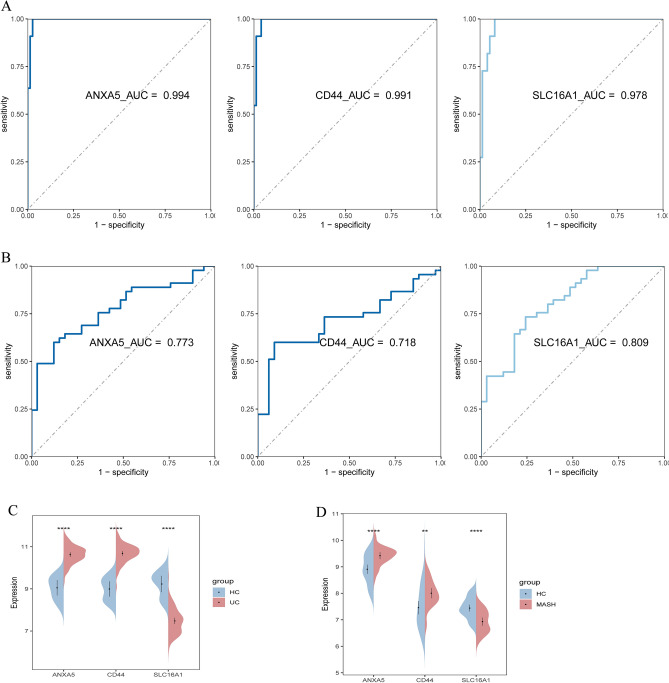
(**A**, **B**) ROC analysis revealing the diagnostic efficacy of 3 sBM-DEGs in the GSE75214 (**A**) and MASH MergeCo (**B**). (**C**, **D**) Expression levels of 3 sBM-DEGs in the GSE75214 (**C**) and MASH MergeCo (**D**).

**Figure 10:**
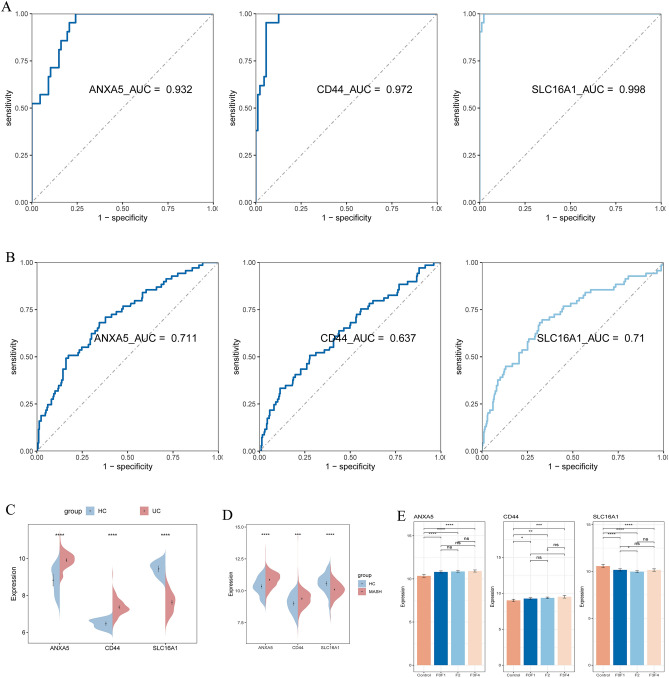
(**A**, **B**) ROC analysis showing the diagnostic efficacy of 3 sBM-DEGs in the GSE87466 (**A**) and GSE213621 (**B**). (**C**, **D**) Expression levels of 3 sBM-DEGs in the GSE87466 (**C**) and GSE213621 (**D**). (**E**) Histogram showing the expression levels of the 3 sBM-DEGs in MASH patients with different degrees of fibrosis.

The expression levels of ANXA5, CD44, and SLC16A1 were validated across multiple datasets: GSE75214, MASH MergeCo, and two external independent datasets (GSE87466 and GSE213621). In both UC and MASH patients, ANXA5 and CD44 were similarly upregulated, while SLC16A1 was downregulated (Figs. 9C,D and Fig. 10C,D).

ROC analysis highlighted the robust diagnostic potential of ANXA5, CD44, and SLC16A1, with AUC values of 0.994, 0.991, and 0.978 in GSE75214, and 0.773, 0.718, and 0.809 in MASH MergeCo, respectively (Fig. 9A,B). The diagnostic accuracy of these markers was further confirmed in the external independent datasets, demonstrating significant efficacy in GSE87466 (AUC: 0.932, 0.972, and 0.998) and GSE213621 (AUC: 0.711, 0.637, and 0.710) (Fig. 10A,B). These findings underscore the compelling diagnostic utility of these three feature biomarkers.

Substantial up-regulation of ANXA5 and CD44 was observed across the F0F1, F2, and F3F4 groups of MASH patients compared to HC (Fig. 10E). Additionally, CD44 exhibited significant expression differences solely between the F0F1 and F3F4 groups. SLC16A1 expression was significantly lower in the F0F1, F2, and F3F4 groups than in the control group, with significant differences between the F0F1 and F2 groups.

**Figure 11:**
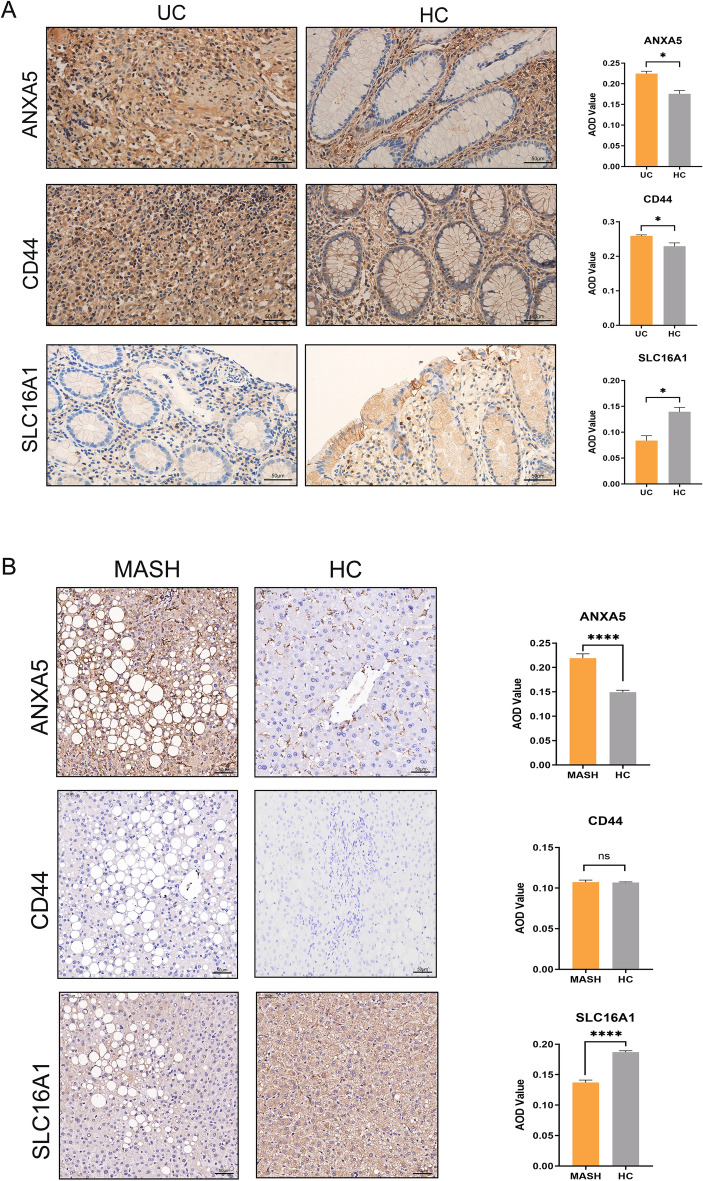
(**A**) Immunohistochemical detection and quantitative analysis of ANXA5, SLC16A1, and CD44 expression in UC patients and HC. Original magnification: 400 × , scale bar = 50 μm. (**B**) Immunohistochemical detection and quantitative analysis of ANXA5, SLC16A1, and CD44 expression in MASH patients and HC. Original magnification: 200 × , scale bar = 50 μm.

Validation of these genes in human UC and MASH samples confirmed these findings. As shown in Fig. 11, ANXA5 was significantly upregulated and SLC16A1 was downregulated in liver tissue of MASH patients and colonic tissue of UC patients. However, CD44 expression showed statistically significant difference only between UC patients and HC.

### Immune cell infiltration analysis

**Figure 12:**
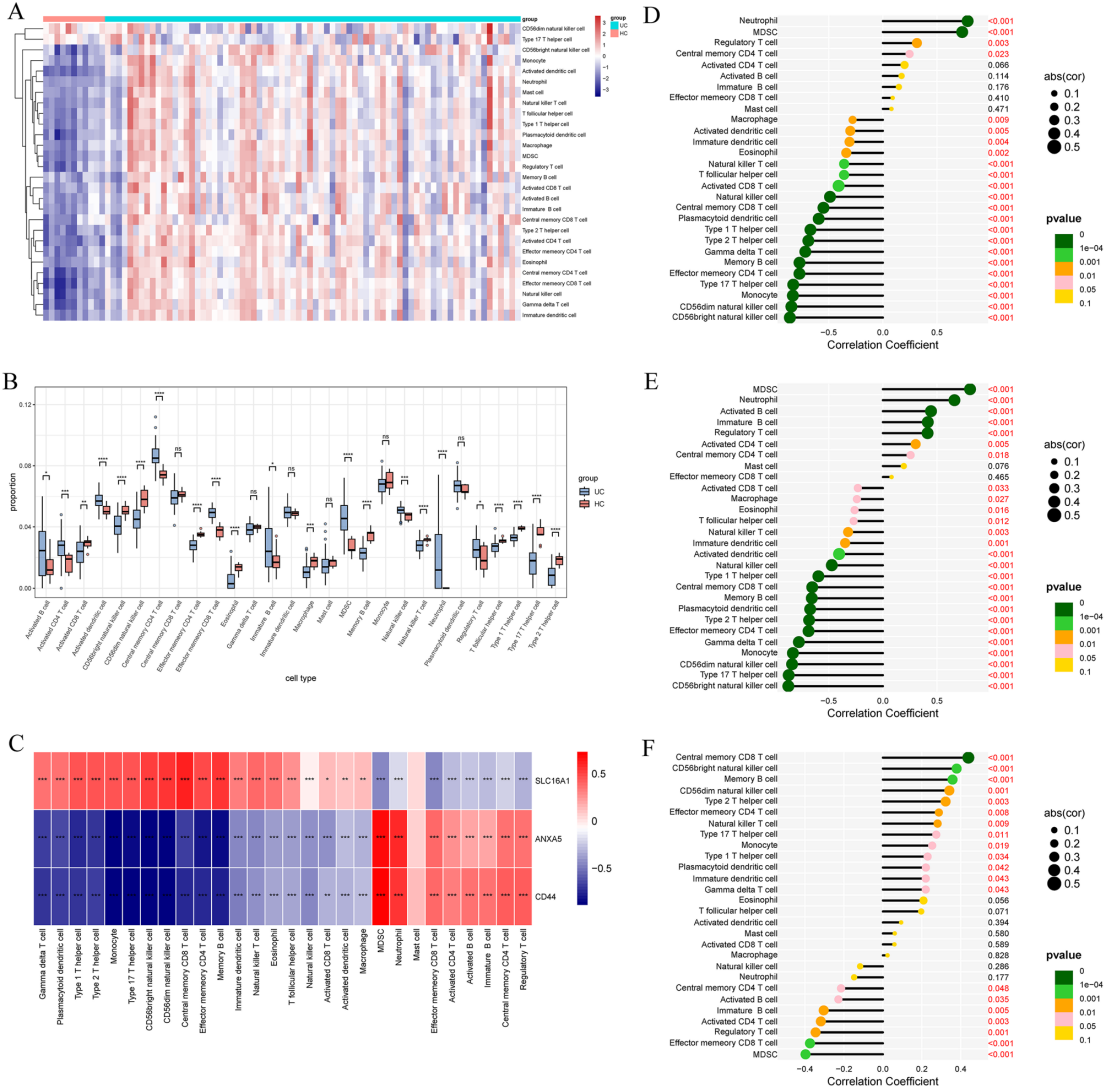
Immune characteristics between UC patients and HC. (**A**) Heatmap depicting the distribution of 28 immune cell infiltrations between UC patients and HC. (**B**) Boxplot revealing differences in infiltrated immune cells between UC patients and HC. (**C**) Heatmap delineating correlations in immune cell infiltrations. (**D**–**F**) Correlation analysis of ANXA5 (**D**), CD44 (**E**), and SLC16A1 (**F**) with immune infiltration.

**Figure 13:**
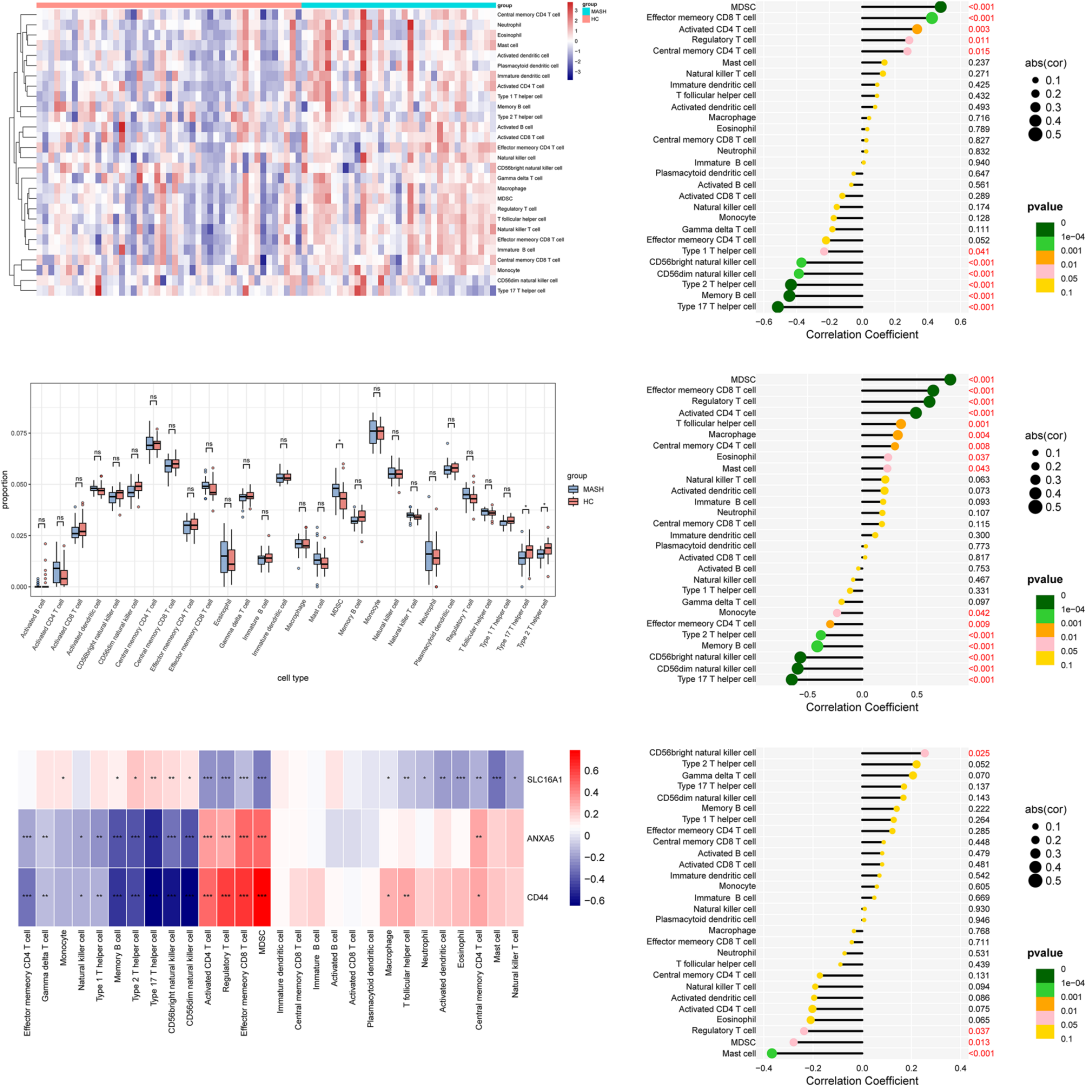
Immune characteristics between MASH patients and HC. (**A**) Heatmap depicting the distribution of 28 immune cell infiltrations between the MASH group and HC. (**B**) Boxplot highlighting differences in infiltrated immune cells between the MASH group and HC. (**C**) Heatmap illustrating correlations in immune cell infiltrations. (D-F) Correlation analysis of ANXA5 (**D**), CD44 (**E**), and SLC16A1 (**F**) with immune infiltration.

The interaction between the three hub genes and the immunological microenvironment was thoroughly investigated. The distribution of 28 distinct immune cell types was assessed in UC (Fig. 12A) and MASH patients (Fig. 13A). UC patients showed significantly elevated proportions of various immune cell populations, including CD56bright natural killer cells, CD56dim natural killer cells, effector memory CD4 T cells, eosinophils, macrophages, memory B cells, natural killer T cells, T follicular helper cells, Type 1 T helper cells, Type 17 T helper cells, and Type 2 T helper cells, compared to HC (Fig. 12B). In MASH patients, elevated proportions of Type 17 T helper cells and Type 2 T helper cells were observed, while myeloid-derived suppressor cells (MDSCs) were reduced (Fig. 13B).

Correlations between the hub genes and the content of immune cells were identified in UC (Fig. 12C) and MASH patients (Fig. 13C). In the context of UC, ANXA5 exhibited a negative correlation with both CD56bright and CD56dim natural killer cells (Fig. 12D). A positive association was observed between CD44 and MDSCs, along with a negative correlation between CD44 and CD56bright natural killer cells (Fig. 12E). SLC16A1 showed a positive association with central memory CD8 T cells and a negative relationship with MDSCs in UC (Fig. 12F). In MASH, ANXA5 and CD44 were positively associated with MDSCs and negatively associated with Type 17 T helper cells (Fig. 13D,E). Additionally, SLC16A1 demonstrated a positive relationship with CD56bright natural killer cells and a negative relationship with mast cells (Fig. 13F). These comprehensive findings illuminate the intricate interactions between the three sBM-DEGs and immune activity.

## Discussion

Accumulating evidence highlighting the robust association between MAFLD and IBD, the specific molecules and mechanisms underlying this association remain elusive^32,33^. Research revealed that decreasing levels of butyric acid could exacerbate damage to the intestinal barrier, affect the liver through leakage through the damaged intestinal barrier, leading to MAFLD^31^. And butyrate produced by *F. prausnitzii* downregulated the pro-inflammatory IL-6/STAT3/IL-17 signal pathway via HDAC1 inhibition and transcriptional repression, while promoting expression of Foxp3 and therefore the maintenance of Treg cells^34^. Indeed, butyrate was found to induce T cell-independent IgA secretion in the colon via activation of GPR41 and GPR109A, and inhibition of histone deacetylase to restore epithelial barrier function under inflammatory conditions. In addition, butyrate had recently emerged as activators of intracellular receptors that control immune responses, such as peroxisome proliferator-activated receptor-γ (PPARγ)^35^. Functional enrichment analysis showed that shared DEGs of MASH and UC patients connected to inflammatory pathways, such as IL-17 signaling pathway, intestinal immune network for IgA production, ECM-receptor interaction, and PPAR signaling pathway. In light of these findings, the mechanism of butyrate metabolism in the co-pathogenesis of MASH and UC was investigated, emphasizing the crucial clinical significance of early recognition and intervention. ANXA5, CD44, and SLC16A1 were identified as shared butyrate metabolism-related DEGs potentially implicated in the co-pathogenesis of MASH and UC.

ANXA5 is a calcium-dependent phospholipid-binding protein that plays diverse roles in cellular processes. By attaching to phospholipids on the surface of apoptotic cells, ANXA5 limits their procoagulant activity and regulates blood coagulation^36^. Additionally, ANXA5 inhibits inflammation, regulates ion channels, and participates in cell signaling pathways^37^. Research suggests that ANXA5 may modulate the inflammatory response in the liver and influence coagulation-related processes during MASH progression^38^. Furthermore, ANXA5's potential impact on intestinal inflammation and mucosal damage in UC has been explored. ANXA5 may regulate inflammatory processes in the gut, contributing to the chronic inflammation characteristic of UC^39,40^.

The CD44 gene, named after its product, the cell surface glycoprotein CD44, plays a key role in cell adhesion, migration, and signal transduction^41^. Beyond its normal physiological functions, CD44 is implicated in various diseases. In patients with MASH, increased CD44 expression is linked to the recruitment of inflammatory cells to the liver, exacerbating liver damage and inflammation^42^. CD44's interaction with hyaluronic acid in the liver's extracellular matrix contributes to fibrosis development, a hallmark of MASH progression^43–45^. Similarly, in UC, CD44 facilitates leukocyte migration and extravasation into inflamed intestinal tissue, intensifying chronic inflammation^46^. CD44's interaction with hyaluronic acid in the gut mucosa is also crucial for epithelial cell repair and tissue regeneration, processes often disrupted in UC patients^47^. Additionally, CD44 isoforms may significantly contribute to both UC and MASH, with dysregulation of specific isoforms associated with disease severity and prognosis, potentially serving as vital biomarkers for disease activity and progression^48^.

SLC16A1, also known as MCT1, is a membrane transporter protein crucial for the transmembrane transport of monocarboxylates such as lactate and pyruvate^49^. It facilitates the uptake of these monocarboxylates generated during glycolysis into cells, where they can be used as an energy source or undergo further metabolism. Additionally, SLC16A1 exports lactate from highly glycolytic cells, preventing intracellular acidification and maintaining optimal pH levels. In the context of MASH, the transport of monocarboxylates such as lactate and pyruvate by SLC16A1 is vital for cellular energy production and waste removal^50^. Dysregulation of SLC16A1 in the liver may lead to metabolic dysfunction and contribute to the progression of MASH^51^. Similarly, in UC, SLC16A1's role in regulating monocarboxylate transport may influence the metabolic and energy balance in inflamed intestinal tissue. Alterations in SLC16A1 expression or activity in UC could impact the availability of energy substrates for intestinal epithelial cells, thereby affecting tissue repair and inflammatory responses^52–54^.

Accumulating evidence demonstrates that both innate and adaptive immune responses are crucial in inducing inflammation in UC and MASH^9^. Bioinformatics results indicate associations between UC, MASH, and various inflammatory and immune pathways. Butyrate significantly modulates adaptive and innate immune responses, regulating T cell differentiation and proliferation. Butyrate enhances Treg cell function, suppresses IL-17 levels, and reduces Th17 cells in the peripheral blood and colon tissues of TNBS-induced colitis rats compared to controls^55^. It inhibits CD4^+^ and CD8^+^ T cell proliferation in a dose-dependent manner and induces apoptosis through the Fas-mediated pathway^56^. Butyrate also promotes Treg cell differentiation by increasing histone H3 acetylation at the FOX3 gene locus^57^. Additionally, it enhances Th1 differentiation by promoting IFN-γ levels and T-bet expression under healthy conditions while inhibiting Th1 differentiation via IL-10 production and T-bet expression during colonic inflammation^58^. This study found that alterations in the abundance of infiltrating immune cells in UC are closely associated with butyrate metabolism-related genes, consistent with previous research. The absence of SLC16A1 impairs CD8^+^ T cell proliferation, shifting ATP production to mitochondrial oxidative phosphorylation. In SLC16A1^*f/f*^T cell ^*cre*^ mice fed a high-fat diet, a reduction in CD8^+^ T cells was observed^59^.

This study successfully identified and validated three key butyrate metabolism-related genes expressed in UC and MASH patients. However, some limitations exist. First, the lack of sequencing data from UC-concomitant MASH patients in the GEO database limited the exploration of gene expression differences between UC and UC-concomitant MASH. Future studies should prioritize expanding the sample size and validating findings across multiple tissue types. Additionally, the mechanistic role of core butyrate metabolism-related genes in the relationship between UC and MASH remains unclear due to the absence of further molecular or animal experiments. Such studies are essential to deepen the understanding of this association.

Overall, this study advanced the understanding of the shared butyrate metabolism-related gene signature underpinning the interplay between UC and MASH. It unraveled potential mechanisms linking the two conditions, highlighting the roles of ANXA5, CD44, and SLC16A1 as pivotal DEGs in butyric acid metabolism between both UC and MASH.

### Supplementary Information

Supplementary Tables.

## Data Availability

These data were derived from the following resources available in the public domain: Gene Expression Omnibus (GEO) database (https://www.ncbi.nlm.nih.gov/geo/query/acc.cgi?acc=GSE61260) (https://www.ncbi.nlm.nih.gov/geo/query/acc.cgi?acc=GSE63067) (https://www.ncbi.nlm.nih.gov/geo/query/acc.cgi?acc=GSE213621) (https://www.ncbi.nlm.nih.gov/geo/query/acc.cgi?acc=GSE87466). Corresponding author can be directed for further inquirements.
