# Supplementary material for: Exploring the butyrate metabolism-related shared genes in metabolic associated steatohepatitis and ulcerative colitis
Source: Sci Rep. 2024 Jul 10;14:15949. doi: 10.1038/s41598-024-66574-0 (PMC11237055; doi:10.1038/s41598-024-66574-0)
Supplement: Supplementary file 1 — Supplementary Tables. [file 41598_2024_66574_MOESM1_ESM.pdf]

Table S1

turquoise_module_genes	blue_module_genes
A2M	A2M
AARS	AARS
ABCC6	ABCC6
ABCD2	ABCC9
ABI2	ABCD2
ABI3	ABHD17C
ABL2	ABI2
ABLIM3	ABI3
ACKR1	ABL2
ACKR3	ABLIM3
ACOD1	ACKR1
ACR	ACKR3
ACSL1	ACR
ACSL4	ACSL1
ACSS3	ACSL4
ACTB	ACSS3
ACTN1	ACTB
ADA	ACTN1
ADAM12	ADA
ADAM23	ADAM12
ADAM28	ADAM19
ADAM8	ADAM8
ADAMTS12	ADAMTS1
ADAMTS3	ADAMTS12
ADAMTS5	ADAMTS2
ADAR	ADAMTS3
ADARB1	ADAMTS5
ADCK5	ADAR
ADCY9	ADARB1
ADGRA2	ADCK5
ADGRE1	ADCY9
ADGRE2	ADGRA2
ADGRE3	ADGRE2
ADGRF5	ADGRE3
ADGRG3	ADGRF5
ADGRG5	ADGRG3
ADGRL2	ADGRG5
ADGRL4	ADGRL2
ADPRH	ADGRL4
AEBP1	ADPGK
AFAP1L1	ADPRH
AGTPBP1	AEBP1
AHR	AGAP2
AIF1	AHR
AIM2	AIF1
AKR1B1	AIM2
AKR7A2	AKNA
AKR7A2P1	AKR1B1
AKR7A3	AKR7A2
AKT3	AKR7A2P1
ALKBH7	AKR7A3
ALOX5AP	AKT3
ALPL	ALDH1L2
AMOTL1	ALKBH7
AMPD2	ALOX5AP
AMPD3	ALPL
AMPH	AMOTL1

ANGPTL2
ANKRD44
ANO2
ANXA2R
ANXA6
AOX1
APBA2
APCDD1
APLNR
APOBEC3F
APOBEC3G
APOL3
ARHGAP24
ARHGAP25
ARHGAP29
ARHGAP30
ARHGAP31
ARHGAP39
ARHGAP45
ARHGAP9
ARHGDIB
ARHGEF16
ARHGEF6
ARL13B
ARL4A
ARL4C
ARRB2
ARSG
ATP10D
ATP13A4
ATP2B4
ATP6V1B2
ATPIF1
AVPR1A
AXIN1
AXL
BACH1
BAIAP2L2
BASP1
BCAR3
BCL2A1
BCL6B
BEST1
BICC1
BIN2
BNC2
BNIP2
BOC
BST2
BTBD9
BTG2
BTK
BTN2A2
BVES
C15orf48
C16orf45
C19orf70
C1orf115

AMPD2
AMPD3
ANGPT2
ANGPTL2
ANKRD44
ANO7
ANTXR1
ANXA2R
ANXA6
AOX1
APBA2
APBB1IP
APCDD1
APLNR
APOBEC3G
APOL3
AQP9
ARHGAP15
ARHGAP24
ARHGAP25
ARHGAP29
ARHGAP30
ARHGAP31
ARHGAP39
ARHGAP9
ARHGDIB
ARHGEF16
ARHGEF6
ARID5A
ARID5B
ARL13B
ARL4A
ARL4C
ARMC7
ARRB2
ARSG
ASAP1
ATF6
ATM
ATP10D
ATP13A4
ATP2B4
ATP5G3
ATP6V1B2
ATP8B2
ATPIF1
AXIN1
AXL
B3GALNT1
BACH1
BAG2
BAIAP2L2
BASP1
BATF
BCAR3
BCAT1
BCL2A1
BCL6

C1orf162
C1orf198
C1R
C20orf194
C21orf91
C2CD2
C3
C3AR1
C5orf52
C9orf72
CACHD1
CACNA2D1
CADM1
CALCRL
CARD10
CARD11
CARD8
CASS4
CAV1
CCDC17
CCDC3
CCDC50
CCDC69
CCDC85C
CCL22
CCL3
CCL4
CCR1
CCR2
CCR4
CD109
CD180
CD2
CD226
CD248
CD28
CD300A
CD300C
CD34
CD37
CD38
CD3E
CD3G
CD4
CD48
CD5
CD53
CD6
CD69
CD79A
CD80
CD81
CD83
CD84
CD86
CD99L2
CDC42SE1
CDH11
BCL6B
BEST1
BGN
BHLHA15
BICC1
BIN2
BLOC1S6
BNIP2
BOC
BST2
BTBD9
BTG2
BTK
BTN2A2
BVES
C15orf48
C16orf45
C16orf54
C19orf70
C1orf115
C1orf162
C1orf198
C1R
C1S
C20orf194
C2CD2
C3
C3AR1
C5AR1
C5orf52
CA5B
CA7
CACHD1
CACNA2D1
CACNB1
CADM1
CALCRL
CALD1
CARD8
CAV1
CBL
CCDC102B
CCDC3
CCDC50
CCDC69
CCDC80
CCDC85C
CCDC88A
CCL3
CCL4
CCR1
CCR2
CCR4
CCR7
CD109
CD180
CD2
CD200

CDH13
CDH5
CDH6
CDK14
CDK17
CDK18
CEBPA-AS1
CEBPB
CECR1
CELF2
CEP120
CEP19
CEP85L
CERCAM
CERS4
CERS5
CFH
CFHR1
CFL2
CFP
CHI3L2
CHN1
CHRDL2
CHST11
CHST15
CLASP1
CLDN23
CLEC14A
CLEC4A
CLEC4D
CLEC4E
CLEC5A
CLEC7A
CLIP4
CLPTM1L
CMKLR1
CMTM2
CMTM7
CNN2
CNPY3
CNRIP1
COL12A1
COL14A1
COL3A1
COL4A1
COL5A1
COL6A1
COL6A2
COL6A3
CORO1A
CPEB4
CREB1
CREB5
CREM
CRIP2
CRISPLD2
CSF1R
CSF2RA
CD226
CD248
CD27
CD28
CD300A
CD300C
CD300E
CD34
CD37
CD38
CD4
CD40
CD48
CD53
CD69
CD79A
CD80
CD81
CD83
CD84
CD86
CD93
CD99L2
CDC42SE1
CDH11
CDH13
CDH5
CDH6
CDK14
CDK17
CDK18
CDS2
CDX1
CEBPA-AS1
CEBPB
CECR1
CELF2
CEP120
CEP128
CEP170
CEP19
CEP85L
CERCAM
CERS4
CERS5
CFH
CFHR1
CFL2
CFP
CHCHD1
CHI3L2
CHMP4B
CHMP5
CHN1
CHRDL2
CHST11
CHST15
CHSY1

CSF2RB
CSF3R
CSGALNACT2
CSRNP1
CST7
CTAGE5
CTHRC1
CTLA4
CTSB
CTSH
CTSK
CXCL12
CXCL13
CXCL5
CXCL6
CXCL8
CXCL9
CXCR2
CXCR4
CXorf36
CYBB
CYFIP2
CYTH4
CYTIP
CYYR1
DAPK2
DCBLD1
DCHS1
DCN
DDR2
DDX24
DEF6
DENND1C
DENND4A
DERA
DERL3
DGAT2
DHRS7B
DKK3
DNAJB9
DNAJC1
DNAJC22
DNM2
DOCK10
DOCK11
DOCK4
DOCK8
DPF2
DPP7
DPYD
DPYSL2
DPYSL3
DSE
DSEL
DTHD1
DUSP14
DYNC2H1
DYSF

CLASP1
CLDN23
CLDND1
CLEC14A
CLEC1A
CLEC2B
CLEC4A
CLEC4D
CLEC4E
CLEC6A
CLEC7A
CLIC2
CLIC4
CLIP4
CLMP
CLPTM1L
CMTM3
CMTM7
CNN2
CNPPD1
CNPY3
CNRIP1
COL12A1
COL14A1
COL15A1
COL1A1
COL3A1
COL4A1
COL4A2
COL5A2
COL6A1
COL6A2
COL6A3
CORO1A
CPEB4
CPNE5
CPVL
CPXM1
CR1
CR1L
CREB5
CREM
CRIP2
CRISPLD2
CRTC3
CSF1R
CSF2RA
CSF2RB
CSF3R
CSGALNACT1
CSGALNACT2
CST7
CTAGE5
CTHRC1
CTLA4
CTSB
CTSH
CTSK

EAF2
EBF3
ECM1
EDNRB
EFEMP2
EGFL6
EGFLAM
EHD1
EHD2
EHD3
ELK4
ELL2
ELMO1
ELOVL5
EMCN
EMILIN1
EMILIN2
EMP3
ENG
ENPP2
ENTPD8
EOGT
EPN3
EPS15
ERMAP
ERO1B
ESRRA
ETFB
ETS1
ETV5
EVI2A
EVI2B
F2R
F5
FAAH
FAM101B
FAM117A
FAM160B1
FAM171A1
FAM171B
FAM20A
FAM20C
FAM216A
FAM234B
FAM30A
FAM46C
FAM49A
FAM49B
FAM53C
FAM65B
FAM78A
FAM83E
FAM89B
FAP
FBLN1
FBLN2
FBLN5
FBN1
CTSL
CXCL10
CXCL12
CXCL13
CXCL5
CXCL6
CXCL8
CXCL9
CXCR1
CXCR2
CXCR4
CXorf21
CXorf36
CYBB
CYLD
CYP7B1
CYR61
CYSLTR1
CYTH4
CYTIP
CYYR1
DAPK2
DBN1
DCBLD1
DCHS1
DCN
DDHD1
DDR2
DDX24
DECR2
DEF6
DENND1C
DENND3
DENND5A
DERA
DERL3
DHRS7B
DKK3
DNAJB9
DNAJC22
DNM2
DOCK10
DOCK11
DOCK2
DOCK4
DOCK8
DOK3
DPF2
DPP7
DPYD
DPYSL2
DPYSL3
DRAM1
DSE
DTX4
DUSP14
DYNC2H1
DYSF

FBXL13
FCER1G
FCGR2B
FCHSD2
FCN1
FCRL5
FERMT3
FFAR2
FFAR3
FGD3
FGD5
FGF7
FGFR1
FGR
FIBIN
FICD
FILIP1L
FKBP11
FLNA
FLRT2
FMNL1
FMNL3
FNDC1
FOXF1
FPR2
FRMD6
FSCN1
FSTL1
FYB
FZD4
G0S2
GAB3
GAS7
GATA6
GBP5
GCA
GFER
GFPT2
GGT5
GIMAP6
GIMAP7
GIT2
GJA4
GJA5
GJC1
GLCCI1
GLI3
GLIPR1
GLT1D1
GLT8D2
GLTP
GLYCTK
GMFG
GNG11
GNS
GOLGA2P5
GPR132
GPR137B
DZIP1
EAF2
ECI1
ECM1
EDNRA
EDNRB
EFEMP1
EFEMP2
EGFL6
EGFLAM
EGR3
EHD2
EHD3
EID1
ELK3
ELL2
ELMO1
ELMO2
ELMO3
ELOVL4
ELOVL5
EMCN
EMILIN1
EMILIN2
EML1
EMP3
ENG
ENPP2
ENTPD1
ENTPD8
EOGT
EPN3
EPS15
ERG
ERMAP
ERO1B
ESAM
ESRRA
ETFB
ETS1
ETV5
EVI2A
EVI2B
F2R
F5
FAAH
FADS1
FAM101B
FAM110B
FAM117A
FAM126A
FAM129A
FAM160B1
FAM171A1
FAM171B
FAM20A
FAM20C
FAM216A

GPR176
GPR183
GPR84
GPRC5B
GPRC5C
GPRIN2
GPT
GPX1
GRAP
GRB10
GRK5
GRK6
GSAP
GUCY1A2
GUCY1A3
GUCY1B3
GVINP1
GYG1
GZMK
HACD4
HAL
HAVCR2
HCAR2
HCAR3
HCK
HEG1
HELB
HEY2
HHEX
HIP1R
HIPK3
HIVEP2
HLA-DPA1
HLA-DPB1
HNF1A
HOOK2
HR
HS3ST3B1
HSD11B1
HSPA13
HSPG2
HTRA1
HTRA3
HYAL2
IER2
IER3
IFI16
IGFBP5
IGFBP7
IGHV5-78
IGKC
IKBIP
IKZF1
IL10RA
IL12RB1
IL17RA
IL18RAP
IL1B
FAM26E
FAM30A
FAM46C
FAM49A
FAM49B
FAM53C
FAM65A
FAM65B
FAM78A
FAM89B
FAP
FAT4
FBLN5
FBN1
FBXL5
FBXL7
FBXW7
FCAR
FCER1G
FCGR2A
FCGR2B
FCGR3A
FCHSD2
FCN1
FCN3
FCRL5
FERMT2
FERMT3
FFAR2
FGD2
FGD3
FGD5
FGF7
FGFR1
FGR
FHL5
FIBIN
FILIP1L
FKBP10
FKBP11
FKBP14
FLI1
FLNA
FLRT2
FLT1
FMNL1
FMNL3
FNDC1
FNDC3A
FOXF1
FPR1
FPR2
FRMD6
FSCN1
FSTL1
FXYD5
FYB
FYN

IL1R1
IL21R
IL27RA
IL2RA
IL2RB
IL33
IL34
IL4R
IL6
IL6ST
IL7R
INPP5D
IQSEC1
IRAK2
IRF4
ITGAL
ITGAM
ITGAX
ITGB2
ITGB7
ITIH5
ITK
ITPKB
ITPRIP
JADE1
JAG2
JAK3
JAM2
JAM3
JAML
JAZF1
JUNB
KAT6A
KATNAL1
KBTBD11
KCNA2
KCNA3
KCNAB2
KCNE4
KCNJ15
KCNK5
KCNN3
KDR
KIAA0040
KIAA0930
KIAA1033
KIAA1211L
KIAA1462
KIAA1551
KIAA1804
KIF12
KIF1C
KIRREL
KLF12
KLF7
KLHDC10
KLHDC9
KLHL12
G0S2
GAB3
GALNT15
GAS7
GATA6
GBP4
GBP5
GCA
GEM
GFER
GFPT2
GGT5
GIMAP4
GIMAP6
GIMAP7
GIMAP8
GIT2
GJA5
GJC1
GK3P
GLCCI1
GLI3
GLIPR1
GLOD5
GLRX5
GLT1D1
GLT8D2
GLTP
GM2A
GMFG
GNB4
GNG11
GNG2
GNS
GOLGA2P5
GPC6
GPR132
GPR137B
GPR141
GPR155
GPR176
GPR183
GPR4
GPR65
GPR84
GPRC5B
GPRC5C
GPRIN2
GPSM3
GPT
GPX1
GPX7
GRAMD1A
GRAP
GRB10
GRB7
GRK3
GRK5

KLHL5
KLHL6
KMO
LAMA4
LAMA5
LAMB1
LAMC1
LAPTM5
LARP6
LAT
LAT2
LAX1
LBH
LCK
LCP1
LCP2
LDB2
LDLRAD3
LEF1
LGALS1
LHFP
LILRA1
LILRA2
LILRA6
LILRB1
LILRB3
LILRB4
LIMD2
LIN7A
LINC00282
LIPN
LIX1L
LMAN1
LMO4
LMTK2
LOC339803
LONRF3
LOX
LOXL1
LRCH1
LRRC25
LRRC32
LRRC57
LRRC8C
LRRK2
LSAMP
LSP1
LTBP3
LY9
MAGEH1
MALT1
MAN1A2
MAP1B
MAP3K12
MAP4K1
MAP4K4
MAP7D3
1-Mar
GRK6
GSAP
GUCY1A3
GUCY1B3
GVINP1
GYG1
GYPC
GZMK
HACD4
HARS2
HAVCR2
HCK
HCLS1
HCST
HECW2
HEG1
HELB
HERC5
HEY2
HGF
HHEX
HIVEP2
HLA-DOB
HLA-DPA1
HNF1A
HOOK2
HOOK3
HR
HRH2
HS3ST3B1
HSD11B1
HSPA13
HSPB8
HTRA1
HTRA3
ICAM1
ICAM2
ICAM3
ICOS
ID2B
IFI16
IFI30
IFNAR2
IGFBP5
IGFBP7
IGFLR1
IGKC
IGSF6
IKBIP
IKZF1
IKZF3
IL10RA
IL12RB1
IL16
IL17RA
IL18RAP
IL1B
IL1R1

MB21D1
MCAM
MCEMP1
MCL1
MCRIP2
MCTP1
MDFIC
MDM2
MEDAG
MEF2C
MEFV
MEI1
MEOX1
MEX3C
MFAP2
MFGE8
MFNG
MFSD14A
MFSD3
MGAM
MGLL
MIEN1
MIR142
MIR200C
MIR223
MISP3
MMP14
MMP2
MMP9
MMRN1
MMRN2
MNDA
MOB3A
MPDZ
MPEG1
MR1
MRAS
MRC2
MRGPRF
MRPL41
MSL3
MSN
MSRB3
MTDH
MX2
MXI1
MXRA7
MYADM
MYCL
MYCT1
MYH10
MYO10
MYO1F
MYO1G
MYO9B
NAGK
NAMPT
NAP1L1
IL21R
IL27RA
IL2RA
IL2RB
IL33
IL34
IL6
IL6ST
IL7R
ILVBL
INPP4A
INPP5D
IQSEC1
IRAK2
IRF4
ISLR
ITGA4
ITGA5
ITGAL
ITGAM
ITGAX
ITGB2
ITGB3
ITIH5
ITK
ITPR1
ITPRIP
JADE1
JAK3
JAM2
JAM3
JAML
JAZF1
KAT6A
KATNAL1
KBTBD11
KCNA3
KCNAB2
KCNE4
KCNJ15
KCNJ8
KCNK5
KCNN3
KCTD20
KDR
KIAA0040
KIAA0930
KIAA1033
KIAA1211
KIAA1211L
KIAA1462
KIAA1468
KIAA1551
KIAA1804
KIF12
KIF1C
KIRREL
KL

NAXD
NCF4
NCKAP1L
NCOA3
NDST2
NDUFA10
NDUFA13
NDUFB7
NEURL1
NEXN
NFATC1
NFATC3
NFE2
NFE4
NFIL3
NFKB2
NFKBIA
NGEF
NID1
NID2
NIN
NINJ1
NIT1
NLGN4X
NLRC4
NLRP3
NNMT
NOD2
NOS3
NOTCH3
NOX4
NPL
NPR1
NR3C1
NR4A3
NR5A2
NRCAM
NRN1
NRP1
NRP2
NUDT16
NUDT16L1
NUGGC
NUP98
NXPE3
OCEL1
OGFRL1
OLFML1
OLR1
OR5B21
ORAI2
OSBPL3
OSBPL8
OSM
P2RX1
P2RX7
P2RY13
P2RY8
KLF7
KLHDC10
KLHDC9
KLHL12
KLHL5
KLHL6
KMO
LAIR1
LAMA4
LAMB2
LAMC1
LAMP3
LAPTM5
LARP6
LAT2
LAX1
LAYN
LBH
LCK
LCP1
LCP2
LDB2
LDLRAD3
LDOC1
LEF1
LGALS1
LHFP
LILRA1
LILRA2
LILRA3
LILRA5
LILRA6
LILRB1
LILRB2
LILRB3
LILRB4
LIMK1
LIX1L
LMAN1
LMTK2
LOC339803
LOC541472
LONRF3
LOX
LOXL1
LOXL2
LPXN
LRCH1
LRCH2
LRRC25
LRRC32
LRRC57
LRRC8C
LRRC8D
LRRK2
LSAMP
LSP1
LST1

P3H1	LUM	
PABPC4	LY6E	
PACSIN2	LY9	
PADI4	LY96	
PAK4	LYVE1	
PALMD	MAGEH1	
PAM	MAGIX	
PAPPA	MAN1A2	
PAPSS1	MAN2B1	
PARD3B	MANEA	
PARP1	MAP1B	
PARVB	MAP3K1	
PCDH12	MAP3K12	
PCDH17	MAP3K3	
PCED1B	MAP4K1	
PCLO	MAP4K4	
PCNX1	MAP7D3	
PCOLCE		1-Mar
PCSK7	MASP1	
PDCD1LG2	MB21D1	
PDE4B	MCAM	
PDGFRA	MCC	
PDK1	MCL1	
PDPN	MCRIP2	
PEA15	MCTP1	
PGPEP1	MDFIC	
PGS1	MDM2	
PHACTR1	MEDAG	
PHC1	MEFV	
PHLDB2	MEI1	
PHTF1	MEX3C	
PHYKPL	MFAP2	
PIAS3	MFGE8	
PIGV	MFNG	
PIK3CG	MFSD14A	
PIK3R3	MFSD3	
PIK3R5	MGAM	
PIKFYVE	MGLL	
PILRA	MGP	
PIM2	MID2	
PINK1	MIEN1	
PKD2	MIGA2	
PLCB2	MIR142	
PLEK	MIR200B	
PLEKHB2	MIR200C	
PLEKHO1	MIR223	
PLEKHO2	MISP3	
PLK2	MIXL1	
PLS3	MLLT11	
PLTP	MMP14	
PLXDC1	MMP19	
PLXNA2	MMP2	
PLXNB1	MMP9	
PLXNC1	MMRN1	
PLXND1	MMRN2	
PNRC1	MNDA	
PODXL	MOB3A	
POU2AF1	MPDZ	

PPARGC1B
PPFIA3
PPIF
PPM1B
PPM1M
PPP1R14C
PPP1R15A
PPP1R16B
PPP3CC
PPT1
PRAF2
PRDM1
PRDM8
PRDX4
PREX1
PREX2
PRICKLE2
PRKAR1A
PRKCB
PRKD1
PRKD3
PROCR
PROK2
PRPS1
PRR14L
PRR16
PRR26
PRRX1
PTAFR
PTGDS
PTGFR
PTGS1
PTGS2
PTPN14
PTPN6
PTPN7
PTPRC
PTPRCAP
PTPRE
PTPRM
PTRF
PYGO1
PYHIN1
QKI
RAB11FIP1
RAB11FIP4
RAB13
RAB29
RAB31
RAB39B
RAC2
RAMP2
RAPGEF4
RASA3
RASAL3
RASGRP3
RASGRP4
RASIP1
MR1
MRAS
MRC2
MRGPRF
MRPL41
MSC
MSL3
MSN
MTDH
MVP
MX2
MXI1
MXRA7
MYADM
MYCBP2
MYCL
MYCT1
MYH10
MYO10
MYO1F
MYO1G
MYO5A
MYO9B
MZB1
NAA35
NABP1
NAGK
NAGPA
NAMPT
NAP1L1
NAXD
NBN
NCF2
NCF4
NCKAP1L
NCKIPSD
NCOA3
NDST2
NDUFA10
NDUFA13
NDUFB7
NDUFS2
NDUFS7
NECAB3
NEURL1
NFATC1
NFKB2
NFKBIA
NFKBIE
NGEF
NID1
NID2
NINJ1
NIT1
NLGN4X
NLRP12
NLRP3
NNMT

RASSF2
RASSF5
RASSF8
RBMS1
RBMS3
RCCD1
RCSD1
RDH13
RECK
RECQL
RERG
RFTN1
RFTN2
RFX5
RGCC
RGS18
RGS2
RHOH
RHOJ
RILP
RIMKLA
RNASE6
RNF149
RNF180
RNF24
ROBO1
ROBO4
ROPN1L
RORA
RPP25L
RSPH3
RUBCN
RUNX3
S100A8
SACS
SAMHD1
SAMSN1
SASH3
SAT1
SCIMP
SCRN1
SDC2
SDK1
SDSL
SEC11C
SEC14L1
SEL1L
SEL1L3
SELL
SELP
SELPLG
SEMA3B
SEMA4A
SEMA4D
SEMA7A
1-Sep
6-Sep
SERPINB9

NOD2
NOTCH3
NOX4
NPR1
NR4A3
NR5A2
NRCAM
NRN1
NRP1
NRP2
NRROS
NT5DC2
NUDT16
NUDT16L1
NUGGC
NUP210
NUP98
NXN
NXPE3
OAZ2
OCEL1
OLFML2B
OR5B21
ORAI2
OSBPL3
OSBPL8
OTUD7B
P2RX1
P2RX7
P2RY13
P2RY8
P3H1
PABPC4
PACS1
PACSIN2
PAK4
PALMD
PAM
PAPPA
PAPSS1
PARD3B
PARVB
PARVG
PCBP3
PCDH12
PCDH17
PCED1B
PCLO
PCNX1
PCOLCE
PCSK7
PDCD1LG2
PDE4B
PDGFRA
PDK1
PDPN
PEA15
PECAM1

SERPINE1
SERPING1
SGCE
SGIP1
SH3BP5
SH3PXD2B
SHC1
SHC3
SIGLEC10
SIPA1L2
SIRPG
SLA
SLAIN1
SLAMF1
SLAMF6
SLAMF7
SLAMF8
SLC12A6
SLC15A3
SLC15A4
SLC1A3
SLC1A4
SLC22A17
SLC22A18AS
SLC26A6
SLC26A8
SLC29A2
SLC2A14
SLC2A3
SLC35B4
SLC38A2
SLC39A4
SLC43A2
SLC7A7
SLC8A1
SLC9A1
SLC9A9
SLC9B2
SLED1
SLFN11
SLFN12L
SLFN5
SMAP2
SMARCA1
SNAI1
SNAI2
SNAPC1
SNCA
SNX10
SNX20
SNX29
SOCS3
SORCS2
SOWAHB
SP100
SP110
SP140
SP4
PEX7
PGPEP1
PHACTR1
PHC1
PHF21A
PHLDB2
PHTF1
PHYKPL
PI15
PIAS3
PIGV
PIK3AP1
PIK3CD
PIK3CG
PIK3R5
PILRA
PIM2
PINK1
PIP4K2A
PKD2
PLA1A
PLA2G7
PLCB1
PLCB2
PLCG2
PLEK
PLEKHB2
PLEKHG3
PLEKHJ1
PLEKHO1
PLEKHO2
PLK3
PLOD1
PLS3
PLTP
PLVAP
PLXDC1
PLXNA2
PLXNB1
PLXNC1
PLXND1
PMP22
PNOC
PNRC1
PODXL
POU2AF1
PPARGC1B
PPFIA3
PPM1B
PPM1M
PPP1R14C
PPP1R16B
PPT1
PRAF2
PRDM1
PREX1
PREX2
PRKAR1A

SPARC
SPARCL1
SPATS2
SPCS3
SPG20
SPN
SPOCK1
SPOCK2
SPON2
SPRYD7
SQRDL
SRGAP2
SRGN
SRPX
ST8SIA4
STAT4
STEAP4
STK4
STX11
STX2
STX3
SULF1
SUMF1
SV2A
SWAP70
SYK
SYNE1
SYT11
SYTL3
SYTL4
SYVN1
TACC2
TACR1
TAF4B
TAGAP
TBC1D10C
TBC1D9
TECPR2
TENM3
TFEC
TFPI
TGFB1
TGFB1I1
TGFB3
TGFBI
TGFBR2
TGIF2
THBS2
THEMIS2
THSD4
THSD7A
TIE1
TIFA
TIMP2
TIMP3
TINF2
TLL1
TLN1
PRKCB
PRKCDBP
PRKCH
PRKD1
PRKD3
PRNP
PROCR
PROK2
PRPS1
PRR14L
PRR16
PRR26
PRRX1
PTAFR
PTGDS
PTGFR
PTGS1
PTGS2
PTPN1
PTPN14
PTPN7
PTPRC
PTPRCAP
PTPRE
PTPRK
PTPRM
PTPRS
PTRF
PXDC1
PXDN
PYGO1
PYHIN1
QKI
RAB11FIP1
RAB11FIP4
RAB13
RAB29
RAB31
RAB39B
RAB8B
RAC2
RAMP2
RAPGEF4
RASA3
RASAL3
RASGRF2
RASGRP1
RASGRP3
RASIP1
RASSF2
RASSF5
RASSF8
RB1
RBMS1
RBMS3
RCCD1
RCSD1
RDH13

TLR1
TLR2
TLR4
TM4SF18
TM6SF1
TMC8
TMEM109
TMEM119
TMEM120A
TMEM154
TMEM156
TMEM164
TMEM204
TMEM246
TMEM255B
TMEM39A
TMEM44
TMEM47
TMEM55A
TMEM64
TMEM88
TMEM8B
TMOD2
TMX3
TNF
TNFAIP8
TNFRSF17
TNFRSF1B
TNFRSF9
TNFSF13B
TNS1
TNS2
TOR1AIP1
TOR3A
TPM2
TPST2
TRAF1
TRAF3IP3
TRAF4
TRAM1
TRAM2
TRAT1
TRBC1
TREM1
TRIM3
TRMT112
TRPC4
TRPC6
TRPS1
TRPV2
TSC22D1
TSHZ3
TSPAN11
TSPAN15
TSPAN18
TSPAN9
TTC39C
TUBG2

RDX
RECK
RECQL
RERG
RFTN1
RFTN2
RFX5
RGCC
RGL1
RGS18
RGS2
RGS5
RHBDD1
RHBDF2
RHOH
RHOJ
RHOQ
RIC1
RILP
RILPL2
RIMKLA
RIPK2
RNASE6
RNF122
RNF144B
RNF180
RNF24
ROBO1
ROPN1L
RORA
RPP25L
RSPH3
RUBCN
RUNX2
RUNX3
S100A12
S100A8
S100A9
S1PR1
S1PR4
SACS
SAMHD1
SAMSN1
SASH3
SCARA3
SCPEP1
SCRN1
SDC2
SDK1
SDSL
SEC14L1
SELL
SELP
SELPLG
SEMA3B
SEMA4A
SEMA4D

1-Sep

TWIST1
TXLNB
TXNDC15
TYROBP
UBAC1
UBE2E2
UBE2J1
UCP2
ULK3
USP11
USP15
USP32
VAMP1
VASH1
VAV1
VCAM1
VCAN
VEGFC
VGLL3
VIM
VKORC1
VNN2
VNN3
VSTM4
VWF
WDR19
WIPF1
WISP1
WNK2
WWP2
XBP1
XPO6
ZCCHC11
ZCCHC24
ZDBF2
ZEB1
ZEB2
ZHX2
ZNF107
ZNF267
ZNF281
ZNF382
ZNF385D
ZNF521
ZNF667-AS1

SERPINB9
SERPINE1
SERPING1
SERPINH1
SERPINI1
SFMBT2
SGCE
SGIP1
SGTB
SH2B2
SH3BP5
SH3PXD2B
SH3RF3
SHANK2
SHC3
SIGLEC9
SIRPA
SIRPB1
SIRPG
SLA
SLAMF1
SLAMF6
SLAMF7
SLAMF8
SLC11A1
SLC15A3
SLC15A4
SLC1A3
SLC1A4
SLC22A18AS
SLC23A2
SLC29A1
SLC29A2
SLC2A14
SLC2A3
SLC35B4
SLC38A2
SLC39A13
SLC39A4
SLC6A6
SLC7A7
SLC8A1
SLC9A1
SLC9A9
SLC9B2
SLFN11
SLFN12
SLFN12L
SLFN5
SMAP2
SMARCA1
SNAI1
SNAI2
SNAPC1
SNCA
SNX10
SNX20

6-Sep

SNX29
SOCS3
SOD2
SORCS2
SOWAHB
SOX5
SP110
SP140
SPAG4
SPARC
SPARCL1
SPATS2
SPCS3
SPG20
SPI1
SPOCK2
SPON2
SPRYD7
SQRDL
SRGAP2
SRGN
SRM
SRPRA
ST3GAL5
ST8SIA4
STAT4
STAT5A
STAT5B
STEAP4
STK10
STK4
STX11
STX2
STX3
SULF1
SUMF1
SV2A
SWAP70
SYK
SYNE1
SYT11
SYTL3
SYTL4
SYVN1
TACC2
TAGAP
TBCEL
TCF4
TDO2
TEK
TFE3
TFEC
TFPI
TGFB1
TGFB1I1
TGFB2
TGFB3
TGFBI

TGFBR2
TGIF2
THBS2
THEMIS2
THSD4
THSD7A
THY1
TIE1
TIFA
TIMP2
TIMP3
TINF2
TLL1
TLN1
TLR1
TLR2
TLR4
TLR6
TLR8
TM4SF18
TM4SF5
TM6SF1
TMEM109
TMEM119
TMEM120A
TMEM154
TMEM156
TMEM164
TMEM204
TMEM243
TMEM246
TMEM255B
TMEM39A
TMEM43
TMEM44
TMEM55A
TMEM64
TMEM71
TMEM8B
TMOD2
TMTC1
TMX4
TNF
TNFAIP2
TNFAIP3
TNFAIP6
TNFAIP8
TNFRSF1B
TNFRSF9
TNFSF13B
TNFSF8
TNS1
TNS2
TOR1AIP1
TOR3A
TPST1
TPST2
TRAF1

TRAF3IP3
TRAF4
TRAM1
TRAM2
TREM1
TRIM10
TRIM22
TRIM3
TRMT112
TRPC1
TRPC4
TRPM4
TRPS1
TRPV2
TSC22D1
TSC22D3
TSHZ2
TSHZ3
TSPAN11
TSPAN15
TSPAN18
TSPAN4
TSPAN9
TSPYL2
TSPYL5
TTC28
TTC39C
TUBA1A
TUBB
TUBG2
TWIST1
TWSG1
TXLNB
TXNDC11
TXNDC15
TYROBP
UBAC1
UBE2E2
UCP2
ULK3
UQCRQ
USP11
USP15
UTRN
VAMP1
VASH1
VAV1
VCAM1
VCAN
VEGFC
VGLL3
VIM
VKORC1
VNN2
VPS37B
VWF
WAS
WDFY4

WDR19
WIPF1
WISP1
WNK2
WWP2
WWTR1
XBP1
ZCCHC24
ZDBF2
ZEB1
ZEB2
ZFP36L1
ZHX2
ZNF215
ZNF267
ZNF281
ZNF366
ZNF385D
ZNF521
ZNF532
ZNF671

Table S2

GeneSymbol	MASH	UC
ACKR2	DN	DN
ACSL4	UP	UP
ALPL	DN	UP
ANXA5	UP	UP
C5AR1	UP	UP
CCL20	UP	UP
CD44	UP	UP
CD53	UP	UP
CHAC1	DN	UP
COL1A1	UP	UP
CXCL10	UP	UP
CXCL13	DN	UP
CXCR2	UP	UP
CXCR4	UP	UP
CYTIP	UP	UP
EVI2B	UP	UP
FCGR2A	UP	UP
GPR183	UP	UP
HLA-DPA1	UP	UP
HPGD	UP	DN
HSD17B11	DN	DN
ITGAX	UP	UP
LAPTM5	UP	UP
LCP1	UP	UP
LRRC31	UP	DN
LYZ	UP	UP
ME1	UP	UP
MEP1B	UP	DN
MGAM	UP	UP
MMP9	UP	UP
MNDA	UP	UP
NCF2	UP	UP
PLXNC1	UP	UP
PRDM1	UP	UP
RGS1	UP	UP
RGS2	UP	UP
S100A8	UP	UP
SELL	UP	UP
SLC16A1	DN	DN
SLC16A4	DN	UP
SLC2A3	UP	UP
SLC51A	DN	DN
SPP1	UP	UP
SRGN	UP	UP
TMEM154	UP	UP
TREM1	UP	UP
UGT2A3	UP	DN
VCAN	UP	UP
VIL1	DN	DN
